# Effects of Ice-Algal Aggregate Export on the Connectivity of Bacterial Communities in the Central Arctic Ocean

**DOI:** 10.3389/fmicb.2018.01035

**Published:** 2018-05-18

**Authors:** Josephine Z. Rapp, Mar Fernández-Méndez, Christina Bienhold, Antje Boetius

**Affiliations:** ^1^HGF-MPG Group for Deep-Sea Ecology and Technology, Alfred Wegener Institute Helmholtz Centre for Polar and Marine Research, Bremerhaven, Germany; ^2^Max Planck Institute for Marine Microbiology, Bremen, Germany; ^3^Fram Centre, Norwegian Polar Institute, Tromsø, Norway; ^4^MARUM – Center for Marine Environmental Sciences, University of Bremen, Bremen, Germany

**Keywords:** sea-ice algae, deep-sea sediment, Illumina tag sequencing, microbial eukaryotes, sea-ice decline, microbial ecology, bacterial diversity

## Abstract

In summer 2012, Arctic sea ice declined to a record minimum and, as a consequence of the melting, large amounts of aggregated ice-algae sank to the seafloor at more than 4,000 m depth. In this study, we assessed the composition, turnover and connectivity of bacterial and microbial eukaryotic communities across Arctic habitats from sea ice, algal aggregates and surface waters to the seafloor. Eukaryotic communities were dominated by diatoms, dinoflagellates and other alveolates in all samples, and showed highest richness and diversity in sea-ice habitats (∼400–500 OTUs). *Flavobacteriia* and *Gammaproteobacteria* were the predominant bacterial classes across all investigated Arctic habitats. Bacterial community richness and diversity peaked in deep-sea samples (∼1,700 OTUs). Algal aggregate-associated bacterial communities were mainly recruited from the sea-ice community, and were transported to the seafloor with the sinking ice algae. The algal deposits at the seafloor had a unique community structure, with some shared sequences with both the original sea-ice community (22% OTU overlap), as well as with the deep-sea sediment community (17% OTU overlap). We conclude that ice-algal aggregate export does not only affect carbon export from the surface to the seafloor, but may change microbial community composition in central Arctic habitats with potential effects for benthic ecosystem functioning in the future.

## Introduction

The Arctic Ocean is one of the marine regions most strongly affected by global climate change, with temperatures currently warming two to three times faster than the global average (Overland et al., 2015). As a result, thick multi-year sea ice is being replaced by thinner first-year sea ice, which only lasts for one melt season (Maslanik et al., 2007; Polyakov et al., 2012). An Arctic Ocean free of summer sea ice has been projected before the end of this century (Johannessen et al., 2004; Overland and Wang, 2013; Notz and Stroeve, 2016). First indications for planktonic community changes associated with Arctic change have been described, including a trend toward reduced eukaryote cell sizes with upper ocean freshening (Li et al., 2009) and shifts in species composition associated with increasing water temperature (Nöthig et al., 2015). Sea-ice decline is likely to increase primary production on the Arctic shelves (Carmack and Chapman, 2003; Arrigo et al., 2008), in regions where enough nutrients are supplied (Tremblay and Gagnon, 2009; Arrigo and van Dijken, 2015; Tremblay et al., 2015a). Increasing light availability in and under thinning ice may also enhance ice-algal productivity and under-ice blooms in the deep ice-covered central basins, which have a lower nutrient availability than the shelves (Lalande et al., 2014; Arrigo and van Dijken, 2015; Fernández-Méndez et al., 2015). Furthermore, the extent of melt ponds in summer is increasing (Rösel and Kaleschke, 2012), opening up new habitats for sea-ice biota (Lee et al., 2011). Recent studies have suggested an important, previously underestimated, role of sea-ice algae in primary production and export flux in the central Arctic Ocean (Assmy et al., 2013; Boetius et al., 2013; Fernández-Méndez et al., 2015). Especially diatoms can form extensive blooms in sea ice, melt ponds and at the bottom of ice floes, and constitute the majority of sea-ice associated biomass (Arrigo, 2014 and references therein). Particularly *Melosira arctica*, which forms long filaments attached to the ice matrix, can build up patchy, but dense accumulations (Melnikov and Bondarchuk, 1987; Fernández-Méndez et al., 2014; Katlein et al., 2014; Poulin et al., 2014). During rapid ice melt, large pulses of these ice algae can be released from the ice (Tamelander et al., 2009) and sink out of the surface ocean, thereby significantly altering the magnitude and composition of organic matter reaching the seafloor (Ambrose et al., 2005; Boetius et al., 2013). Yet, the low number of documented observations of such large ice algae export events and the relatively poor knowledge of spatial and temporal variability of microbial community composition in the central Arctic currently impede predictions about the effects of such environmental changes on biodiversity and ecosystem functioning (Galand et al., 2010; Comeau et al., 2011; Wassmann, 2011; Ghiglione et al., 2012; Thaler and Lovejoy, 2015).

In the Arctic Ocean, in contrast to other ocean environments, temperatures close to the freezing point prevail across sea ice, water column and seafloor habitats, potentially permitting a close vertical connectivity of communities. Seed communities in transient environments such as sea ice are likely derived from directly connected environments (Ackley and Sullivan, 1994), i.e., surface ocean waters and sediments that are incorporated during ice formation on the shallow Arctic shelves (Wegner et al., 2017). In addition, the focused seasonal particulate organic carbon flux from the sea ice and surface ocean to the seafloor may transport viable microbes to the seafloor, where they could contribute to the turnover of carbon and nutrients, and potentially also become members of the benthic communities (Turley and Mackie, 1995; Ruff et al., 2014).

This study aimed at assessing the community composition and turnover of bacterial and eukaryotic microorganisms associated with ice-associated and sinking algal aggregates, as well as their similarity with potential source communities of sea ice, water and deep-sea sediments using next-generation sequencing. We hypothesized (i) that sea-ice algae aggregates select for specific members of eukaryotes and bacteria; (ii) that these originate from sea ice rather than the water column; (iii) that these are exported by rapidly sinking aggregates that transport sea-ice life to the deep sea, but (iv) get quickly overgrown by sediment microbes. Furthermore, this study contributes baseline knowledge of microbial diversity in the central Arctic Ocean in times of rapid sea-ice melt.

## Materials and Methods

### Study Site and Environmental Conditions

**FIGURE 1:**
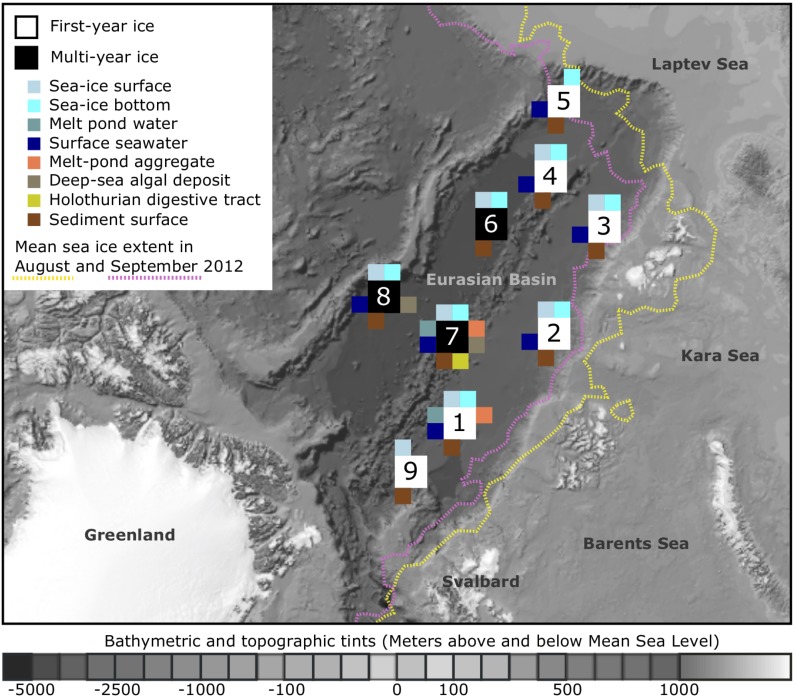
Sampling locations and sampled environments. Map of the central Arctic Ocean, depicting sampling locations and mean sea-ice extent during RV Polarstern expedition ARK-XXVII/3 (PS80) in 2012. A total of nine stations were visited from August to the end of September, with the individual sampled environments indicated by color. The bathymetric map was modified from Jakobsson et al. (2012). For further details on sampling location and the conducted analyses see Supplementary Table S1.

Our samples were retrieved from nine sampling locations across the Eurasian Basin of the central Arctic Ocean during RV *Polarstern* expedition ARK-XXVII/3 (PS80) in August and September 2012 at the end of the productive season (**Figure 1**). Surface seawater temperatures were between -1.8 to -1.5°C and salinity ranged from 30.3 to 33.2 (Boetius et al., 2013). Nutrient inventories in the surface layer were already very low and nitrate generally limiting (Fernández-Méndez et al., 2015). First-year ice was the dominant ice type during the time of sampling (Supplementary Table S1), and ice thickness varied between 0.7 and 2 m (Fernández-Méndez et al., 2014). Salinity of the total sea-ice surface ranged between 0.1–2.9, and 1.0–3.6 in the bottom layer. Temperatures within surface sea ice varied from -8.5 to 0°C and were between -1.9 and -1.1°C in bottom ice (Hardge et al., 2017). Melt-pond coverage on ice floes varied between stations and ranged from 20 to 50%. Different types of melt ponds were observed, including closed (closed to the underlying seawater), and open ponds (directly connected to the underlying seawater). Some of them had a refrozen surface ice cover. Sea-ice algal aggregates of varying size and color were observed in all different types of ponds (Fernández-Méndez et al., 2014). Seafloor observations revealed the presence of deposited ice-algal aggregates on the sediment surface in the deep sea, with an especially high carbon deposition of 32 and 156 g C m^-2^ at stations Ice7 and Ice8 (**Figure 1**), when compared to the remaining stations where values ranged from 0 to 20 g C m^-2^ (Boetius et al., 2013). The presence of algae deposits at the seafloor often coincided with the presence of opportunistic holothurians of the species *Kolga hyalina*, which were attracted by the fresh food source (Boetius et al., 2013). Bottom water temperatures in the deep sea were stable at around -0.7°C (Rabe et al., 2013). Direct links to the original data on environmental conditions during sampling can be found via the PANGAEA database entry (Rapp et al., 2017).

### Sampling

Sea-ice algal aggregates and melt pond water were sampled from three distinct melt ponds at stations Ice1 and Ice7 (**Figure 1** and Supplementary Table S1) using a manually operated vacuum pump (Model 6132-0010, Nalgene, Penfield, NY, United States). Deposited ice-algal aggregates from the deep-sea floor at stations Ice7 and Ice8 were retrieved using a TV-guided multicorer or a MultiGrab, and individually collected from the sediment surface with sterile plastic pipets or forceps. Sea-ice cores from all stations were taken with an ice corer (9 cm diameter) (Kovacs Enterprise, Roseburg, OR, United States), cut into two equal sections and melted in plastic containers (previously rinsed with ethanol and ultrapure water) on a shaker in the dark at 4°C (Mikkelsen et al., 2008; Rintala et al., 2014). Ice core length ranged from 0.8 to 2.0 m (Supplementary Table S1). Four ice samples were not sectioned: two ice cores from station 9 (one of them from newly formed ice and the other one from very thin ice adjacent to it), as well as two cores from station 7 and 8, which showed a strong brownish coloration (indication of high algal biomass) (Supplementary Table S1). Melting of the ice took around 24 h and samples were immediately processed as soon as the last piece of ice melted, to avoid abrupt changes of temperature in the sample. Surface seawater was sampled either with a peristaltic pump through a hole in the sea ice (Masterflex^®^ E/STM portable sampler, 115 VAC, Oldham, United Kingdom), or with a conductivity–temperature–depth (CTD) rosette sampler (Seabird SBE 911 plus, Bellevue, WA, United States) at 0–2 m depth. Between 0.5 and 2 L of melted sea ice or water were filtered through 0.22 μm polyethersulfone membranes (Millipore^®^ Sterivex^TM^, Merck KGaA, Darmstadt, Germany) with a multichannel peristaltic pump (Model PD 51; Heidolph, Schwabach, Germany). Three parallel deep-sea sediment cores were retrieved from each sampling site by a TV-guided multicorer and subsamples of the uppermost centimeter of each core were pooled per site. At station Ice1, we obtained a total of nine sediment cores, allowing us to prepare three replicate sediment samples (Supplementary Table S1). Holothurians feeding on the deposited algal aggregates were collected using an Agassiz trawl, digestive tracts were dissected and the content stored in plastic syringes. All Sterivex filters and deep-sea samples were stored at -20°C until further processing.

A total of 54 samples were taken for microbial community analysis, 52 of these were subject to bacterial community analysis, and 45 of these samples to eukaryotic community analysis (Supplementary Table S1).

### DNA Extraction, PCR and Illumina Sequencing

Total community DNA was extracted from half Sterivex filters for sea-ice, melt-pond and seawater samples, from 0.5 g of algae aggregates and holothurian digestive tract content and from 1 g of sediment using the UltraClean^TM^ Soil DNA Isolation Kit (MoBio Laboratories, Inc., Carlsbad, CA, United States) following the manufacturer’s instructions for maximum yields, with minor modifications. Instead of using the kit’s enclosed solution S5, DNA extracts were eluted in a final volume of 80 μl TE-buffer (10 mM Tris-Cl, pH 8.0; 1 mM EDTA). By using one standardized extraction kit across all sample types, we aimed to minimize potential biases toward differential extraction yields for individual microbial taxa and thus increase comparability. DNA concentration and purity were determined by fluorescence-based Qubit^®^ quantitation assays (Life Technologies GmbH, Darmstadt, Germany). Amplicon libraries of the bacterial V4-V6 region of the 16S rRNA gene and the eukaryotic V4 region of the 18S rRNA gene were generated according to the protocol recommended by Illumina (16S Metagenomic Sequencing Library Preparation, Part #15044223, Rev. B). For selected sediment, sea-ice algae and deposited algae samples we obtained technical replicates, by preparing several sequencing libraries from the same DNA extract (Supplementary Table S1). For *Bacteria* we selected the S-D-Bact-0564-a-S-15 and S-^∗^Univ-1100-a-A-15 primer pair based on a primer evaluation by Klindworth et al. (2013) and for *Eukaryota* the TAReukFWD1 and TAReukREV3 primers (Stoeck et al., 2010) (Supplementary Information Scripts bac and euk). Libraries were sequenced on an Illumina MiSeq platform in 2x300 cycles paired end runs. Raw paired-end sequences have been submitted to ENA under INSDC accession number PRJEB23005 using the data brokerage service of the German Federation for Biological Data (GFBio, Diepenbroek et al., 2014), in compliance with the Minimal Information about any (X) Sequence (MIxS) standard (Yilmaz et al., 2011). To corroborate the observed bacterial community patterns, we used Automated Ribosomal Intergenic Spacer Analysis in parallel to the tag sequencing approach for a wider set of in total 68 samples (Supplementary Information Material and Methods).

### Sequence Processing, Taxonomic Assignment and Data Filtering

An initial 10,266,909 bacterial and 3,717,438 eukaryotic raw sequence pairs were generated (Supplementary Table S2). We used *cutadapt* (v. 1.8.1; Martin, 2011) for the removal of primer sequences and a custom *awk* script to ensure the correct orientation of reads prior to merging (Supplementary Information Scripts bac and euk). For merging forward and reverse reads we used *pear* (v. 0.9.5; Zhang et al., 2014) and trimmed and quality filtered all sequences using *trimmomatic* (v. 0.32; Bolger et al., 2014). 2,225,003 bacterial and 2,777,311 eukaryotic merged and quality trimmed sequences were retained after processing (Supplementary Table S2). We reassured correct formatting of the fastq files with bbmap (v. 34.00; B. Bushnell^1^) before clustering the reads into 613,270 bacterial and 59,853 eukaryotic OTUs by applying a local clustering threshold of *d* = 1 and the fastidious option in *swarm* (v. 2.1.1; Mahé et al., 2015). After alignment with the SINA aligner (v. 1.2.10; Pruesse et al., 2012) and classification of the seed sequence of each OTU with the SILVA SSU database release 123 (Quast et al., 2013), we removed all OTUs that were classified as chloroplasts, mitochondria, archaea, or those that could not be classified at domain level from further analysis. OTUs that were classified as bacteria within the eukaryotic dataset and vice versa, were removed as well. Furthermore, we removed all absolute singletons, i.e., OTUs that were only represented by a single sequence across the complete dataset (Supplementary Table S2). By doing so we reduced the effect of artificially inflated diversity potentially introduced through sequencing errors (Tremblay et al., 2015b). Filtering and removal of absolute singletons resulted in a final number of 8,869 bacterial and 7,627 eukaryotic OTUs (Supplementary Table S2). All further analyses were performed with these processed OTU abundance tables (Rapp et al., 2017). The full bioinformatic scripts are provided as supplementary information (Supplementary Information Scripts bac and euk).

### Community Comparison and Analysis of Abundant and Dispersed Taxa

We calculated OTU numbers, as well as the Chao1 richness estimate and the inverse Simpson diversity index per sample. To obtain reliable estimates independent of differences in sequencing depth between samples, we subsampled our input OTU tables (Rapp et al., 2017) 100-times to the lowest read number of each dataset (bacteria: 10,068 reads; eukaryotes: 16,372 reads) and used mean values across all iterations. Significant differences in OTU numbers between habitats were determined by analysis of variance using permutation tests as implemented in the function *aovp* from the *lmperm* package (Wheeler and Torchiano, 2016). *Post hoc* pairwise permutation tests were performed using the function *pairwisePermutationTest* from the *rcompanion* package (Mangiafico, 2017), and p-values were corrected for multiple testing using the Benjamini–Hochberg procedure (Benjamini and Hochberg, 1995). We generated species accumulation curves at different taxonomic resolution to assess the efficiency of our sampling effort to capture the present diversity in the individual environments (Supplementary Figures S1–S4). To assess overall differences in community structure we calculated Bray-Curtis dissimilarity matrices and visualized the results in non-metric multidimensional scaling (NMDS) plots. Due to the high variability in 18S gene copy numbers of microbial eukaryotes, which can vary by at least four orders of magnitude, depending on genome (Prokopowich et al., 2003) and cell (Zhu et al., 2005) size, we additionally assessed community structure using the Jaccard dissimilarity measure for presence/absence of eukaryotic taxa (Supplementary Figure S5). Correlation of both dissimilarity measures was examined by performing a mantel test with 999 permutations (Supplementary Figure S6). Concomitantly, we performed an analysis of similarity (ANOSIM) to test for the significance of differences between groups of samples from different environment types. All analyses were conducted in R (v. 3.1.2; R Core Team, 2014) using the *vegan* package (Oksanen et al., 2014) and custom R-scripts (Supplementary Information Scripts bac and euk; Gobet et al., 2010). For further community comparisons we chose a conservative presence-absence approach. We aimed to distinguish between abundant, widely dispersed groups of an environment and transient groups, which are present at a subset of locations (Shade and Handelsman, 2012). Therefore, we applied a rule that would count a taxon (i.e., an OTU) as dispersed and abundant, if found in >50% of the samples of a habitat, and represented by at least 100 sequences (Supplementary Tables S3, S4). Next, we determined the proportion of community overlap (i.e., shared groups) between the sampled environments using Jaccard similarities. Further, we defined those OTUs that were shared across sea ice, water column, aggregates, as well as deep-sea surface sediments as generalists. Consequently, habitat-specific OTUs are members of only one environmental community.

## Results

### Richness and Diversity of Microbial Communities in Central Arctic Ocean Habitats

**FIGURE 2:**
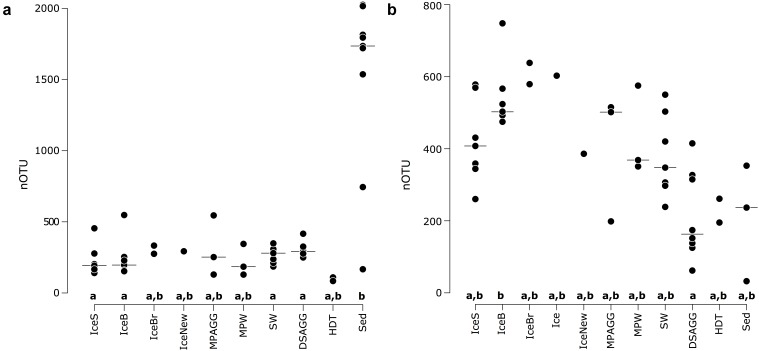
Number of OTUs for **(a)** bacterial communities and **(b)** eukaryotic communities, with the horizontal black line indicating the median value per environmental grouping. Lower case letters at the bottom of the figure indicate environments that are significantly different from each other based on ANOVA permutation tests at a significance threshold of *p* < 0.05. IceS, sea-ice surface; IceB, sea-ice bottom; IceBr, brown sea ice; Ice, sea ice; IceNew, freshly formed sea ice; MPAGG, melt-pond aggregate; MPW, melt-pond water; SW, surface seawater; DSAGG, deep-sea algae deposit; HDT, holothurian digestive tract content; Sed, deep-sea surface sediment.

We saw indications for higher eukaryotic and bacterial community richness in sea-ice algal aggregates than in the surrounding melt-pond water, and in the case of bacteria, also than in adjacent sea ice (**Table 1** and **Figure 2**). Only 11–12% of the OTUs associated with ice-algae aggregates met our definition of dispersed, abundant community members, i.e., they were present in >50% of aggregate samples and represented by at least 100 sequences. These OTUs represented the majority of sea-ice algae associated sequences (**Table 1**). Melt-pond waters showed the lowest eukaryote and bacterial OTU richness of the sampled ice habitats, but had a relatively large fraction of abundant bacterial types (23%). Bacterial communities in sea ice also exhibited relatively low richness, while eukaryotic communities showed highest richness in sea-ice samples (**Figure 2**).

**Table 1:** Community alpha diversity and contribution of abundant and dispersed community members.

	Bacteria								Eukaryota							
	IceS	IceB	MPW	MPAGG	SW	DSAGG	HGC	Sed	IceS	IceB	MPW	MPAGG	SW	DSAGG	HGC	Sed
	*n* = 7	*n* = 7	*n* = 3	*n* = 3	*n* = 7	*n* = 7	*n* = 2	*n* = 9	*n* = 7	*n* = 8	*n* = 3	*n* = 3	*n* = 7	*n* = 8	*n* = 2	*n* = 3
nOTUs ^m^	192	196	185	252	280	292	97	1,736	408	503	369	502	348	163	228	237
Total OTUs	1,374	1,457	657	1,016	874	1,446	173	5,612	2,576	3,545	2,095	1,742	1,730	1,413	435	497
SSOs ^m^ [%]	20	18	17	16	17	14	14	42	1	3	2	2	2	1	1	2
Chao1 ^m^	265	307	269	343	369	441	111	2,415	684	916	747	833	449	239	277	242
invS ^m^	9	9	4	10	22	10	3	154	9	15	17	5	11	3	9	7
A&D OTUs	149	138	148	117	204	190	33	413	208	284	240	184	228	109	111	61
A&D fraction	11	10	23	12	23	13	19	7	8	8	12	11	13	8	26	12
Total reads	249,151	216,935	103,504	62,740	271,203	194,048	36,786	171,767	400,528	462,135	239,671	207,779	391,252	398,697	132,294	102,951
A&D reads	235,827	197,078	98,772	52,935	258,915	184,516	35,105	118,626	377,330	422,583	224,213	194,520	357,325	371,597	128,930	72,378
A&D read fraction	95	91	95	84	96	95	95	69	94	91	94	94	91	93	98	70

*nOTUs, OTUs present in an individual sample; total OTUs, the sum of unique OTUs detected in a given environmental grouping; SSOs, singleton OTUs, represented by only a single sequence across the total dataset, removed from analysis prior to alpha diversity calculation; Chao1, chao1 richness estimate; invS, inverse Simpson diversity index; A&D community, abundant and dispersed OTU present in >50% of the samples within an environmental grouping and represented by at least 100 sequences across the dataset; A&D fraction, proportion of abundant and dispersed OTUs relative to the total number of OTUs; Total reads, the sum of all reads after sequence processing. IceS, sea-ice surface; IceB, sea-ice bottom; MPW, melt-pond water; MPAGG, melt-pond aggregate; SW, surface seawater; DSAGG, deep-sea algae deposit; HDT, holothurian digestive tract content; Sed, deep-sea surface sediment. m, median values per environmental grouping.*

**FIGURE 3:**
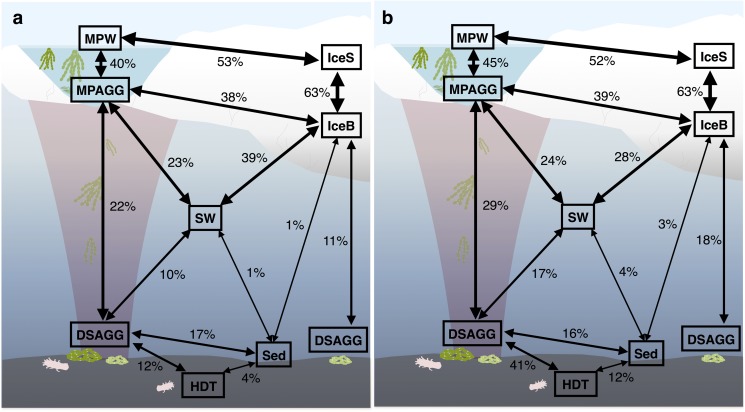
Community overlap between environments for **(a)** bacteria and **(b)** eukaryotes. Shared proportions are based on presence-absence data for abundant and dispersed community OTUs only, including these OTUs that were present in at least 50% of the samples from one environment and represented by at least 100 sequences in the dataset. IceS, sea-ice surface; IceB, sea-ice bottom; MPAGG, melt-pond aggregate; MPW, melt-pond water; SW, surface seawater; DSAGG, deep-sea algae deposit; HDT, holothurian digestive tract content; Sed, deep-sea surface sediment.

Bacterial communities on sunken ice-algal aggregates deposited at the seafloor (4,400 m water depth) during the summer season were slightly richer than the communities on photosynthetically active aggregates in melt ponds, communities in sea ice and in surface seawater. They still shared 22% of their abundant OTUs with the original ice-algae associated community, but also 17% with the abundant, dispersed sediment bacteria (**Figure 3a**). We derived the relative contribution of these shared OTUs (Supplementary Table S3) to the total communities detected in deposits and sediments (Rapp et al., 2017). The OTUs shared between sediments and deposits accounted for on average 55% of the bacterial sequences in the algal deposits (up to 79% in individual aggregates), but only for 28% of sequences in the total sediment community. In contrast, eukaryotic communities of the deposited algae showed lowest richness and diversity compared to all other environments (**Table 1** and **Figure 2**). They shared only 29% with the algae-associated abundant types (**Figure 3b**), and 16% with the abundant sediment eukaryotes. Bacterial richness and diversity was lowest in the digestive tract content of holothurians feeding on the deposited aggregates on the seafloor (**Table 1** and **Figure 2**), and these bacteria were mostly unique to the holothurian digestive tract, with only 4% overlap with the abundant, dispersed types of the sediment community (**Figure 3**). Deep-sea surface sediments contained a significantly richer and more diverse bacterial community than sea ice, seawater and algae deposits, and showed almost no overlap with water column and sea-ice communities, while eukaryotic richness in sediments was at the lower end of the observed range across all sampled environments (**Figure 2**).

**FIGURE 4:**
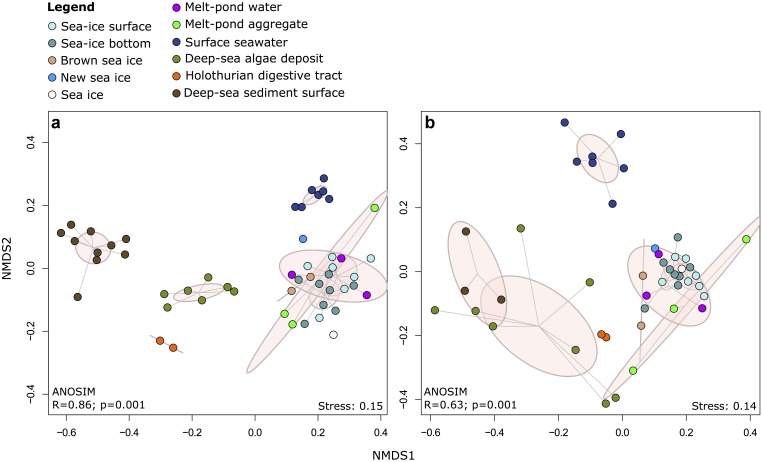
Two-dimensional NMDS ordination of community dissimilarities for **(a)** bacteria and **(b)** eukaryotes. Dissimilarity matrices and ANOSIM were calculated using the Bray-Curtis dissimilarity measure on the basis of relative abundances of Illumina OTUs. Environments are depicted by color coding and points within each environmental grouping are connected to their group centroid through a spider diagram. Pink ellipses indicate the estimated 95% dispersion limits of each group.

Non-metric multidimensional scaling (NMDS) of Illumina OTUs clustered the samples into five groups according to their habitat (**Figure 4** and Supplementary Figure S5), including communities from (i) sea-ice associated environments (sea-ice algae aggregates, melt ponds and sea ice), (ii) surface seawater, (iii) deep-sea sediment, (iv) deposited sea-ice algal aggregates and (v) holothurian digestive tract both for bacteria (ANOSIM: *R* = 0.86, *p* = 0.001) and eukaryotes (ANOSIM: *R* = 0.63, *p* = 0.001). In both datasets, the communities in sea-ice algal aggregates showed high within-group variation. Notably, eukaryotic community structure in deep-sea sediment and exported ice-algal aggregates showed a much larger dissimilarity than bacterial communities (**Figure 4b**). Overall, the dissimilarities of bacterial and eukaryotic community structures were significantly correlated across all habitats (*r* = 0.76; *p* = 0.001) (Supplementary Figure S7). Bacterial community patterns observed with Illumina tag sequences were supported by analyses of ARISA patterns, which built on a larger set of samples, i.e., from melt pond water, sea-ice algae aggregates and holothurian digestive tract (Supplementary Figure S8); dissimilarity matrices from both methods were significantly correlated (Supplementary Figure S9 and Supplementary Information Results).

### Composition of Microbial Communities in Sea Ice, Surface Water and Sediments

**FIGURE 5:**
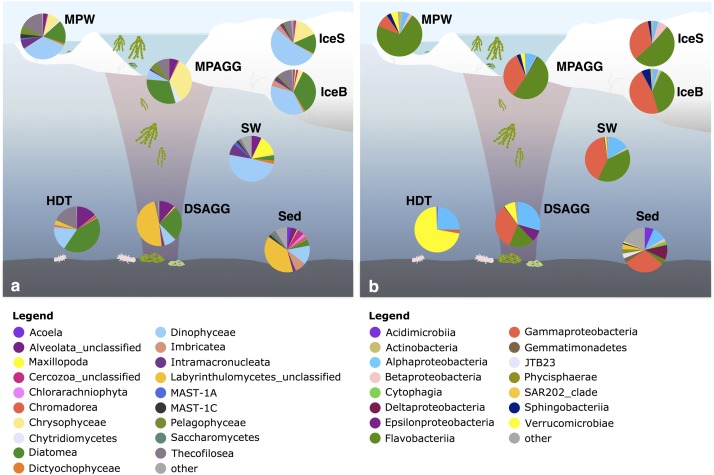
Dominant **(a)** eukaryotic and **(b)** bacterial classes in the central Arctic Ocean. Shown are average values per environment, displaying classes with a relative sequence abundance of at least 2%. The remaining classes were grouped as ‘other.’ IceS, sea-ice surface; IceB, sea-ice bottom; MPAGG, melt-pond aggregate; MPW, melt-pond water; SW, surface seawater; DSAGG, deep-sea algae deposit; HDT, holothurian digestive tract content; Sed, deep-sea surface sediment. For details on the samples, see Supplementary Table S1.

**FIGURE 6:**
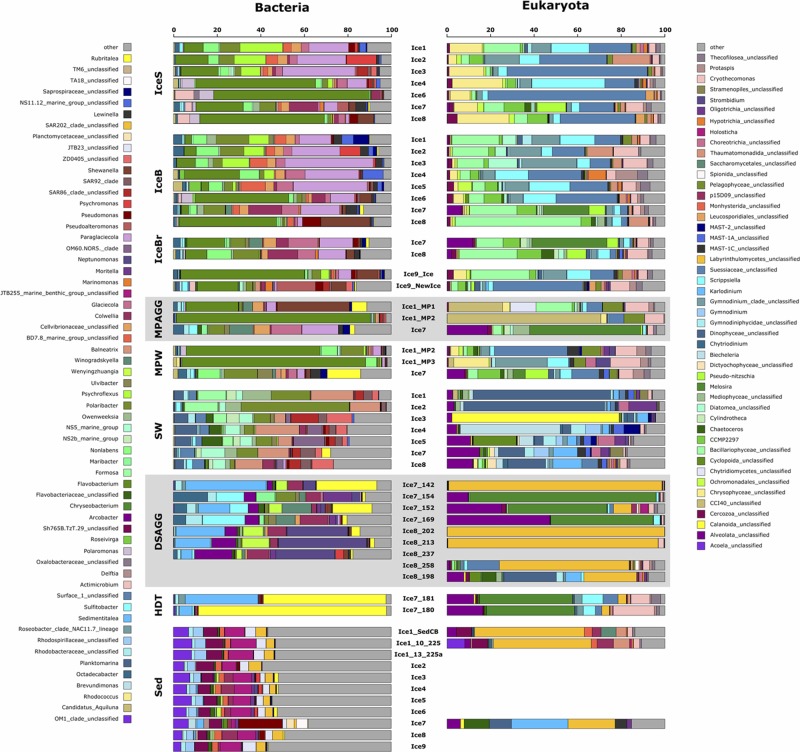
Dominant bacterial and eukaryotic genera in a range of environments across the Eurasian basin of the Arctic Ocean. Shown are the five most abundant genera across all samples, based on relative sequence abundance. Samples are grouped by environment and corresponding samples from the bacterial and eukaryotic dataset are plotted next to each other. IceS, sea-ice surface; IceB, sea-ice bottom; IceBr, brownish-colored sea ice; MPAGG, melt-pond aggregate; MPW, melt-pond water; SW, surface seawater; DSAGG, deep-sea algae deposit; HDT, holothurian digestive tract content; Sed, deep-sea surface sediment.

At the time of sampling in late summer, contribution of diatom sequences in surface seawater was minor (**Figure 5a**). *Dinophyceae* were the predominant group in surface seawater, but the majority of its representatives could not be classified (**Figure 6**). One dominant genus was *Karlodinium*, which was also observed in individual deposited algae aggregates at the seafloor, and in very high sequence abundance in Ice7 deep-sea sediment, where we observed high algae deposition. Eukaryote community composition in sea ice comprised green-brown algae and *Dinophyceae*, mainly the genus *Scrippsiella*, members of the Gymnodinium clade and unclassified *Suessiaceae* in the upper part of the cores, and mostly unclassified diatoms at the bottom of the ice (**Figure 6**). The genera *Pseudo*-*nitzschia* and *Melosira* exhibited a patchy distribution with low relative abundance (<1%) at all stations except for Ice7. All of these groups were also present in melt-pond waters, in addition to other algae from the *Pelagophyceae* and a larger contribution of *Thecofilosea*, including the nanoflagellate *Cryothecomonas*. In deep-sea sediments, Labyrinthulomycetes, dinoflagellates and other flagellates from the *Imbricatea* dominated eukaryote communities (**Figures 5a**, **6**). At Ice7 we observed a relatively high contribution of the diatom *Chaetoceros*, the only diatom detected in the sediment outside of the algal deposits.

*Flavobacteriia*, *Gamma*- and *Alphaproteobacteria* dominated bacterial communities in sea ice, surface seawater and sediments (**Figure 5b**), yet with pronounced differences in their predominant representatives at the genus level (**Figure 6**). In sea ice and melt ponds, *Flavobacteriia* were mainly represented by the genus *Flavobacterium*, with reads of this single genus constituting 9–71% of the total community in the ice surface and 3–45% in the bottom of the ice. Also the genera *Polaribacter*, *Psychroflexus*, *Nonlabens*, and *Ulvibacter* were sequence- abundant in ice. Among these, *Polaribacter* was the only genus, which was also a dominant flavobacterial member in surface seawater. Here, the genera *Owenweeksia*, *Formosa* and the NS5 marine group were the most sequence-abundant *Flavobacteriia* (**Figure 6**). Microbial community composition of the deep-sea floor was quite distinct from the water column, and the contribution of *Flavobacteriia* was markedly reduced (**Figure 5b**). Most of its representatives in surface sediments could not be classified at genus level and clustered as unclassified *Flavobacteriaceae*.

*Gammaproteobacteria* in sea ice were dominated by *Paraglaciecola*, which displayed a similarly large variability in its contribution as *Flavobacterium*, ranging from 0 to 33% in sea-ice surface communities and 2–40% in bottom communities (**Figure 6**). Other important *Gammaproteobacteria* in sea ice belonged to the genera *Colwellia* and *Glaciecola*. In melt-pond waters *Gammaproteobacteria* representation was lower (**Figure 5b**), but *Paraglaciecola* and *Glaciecola* were also dominant members (**Figure 6**). In the water column, the most prominent *Gammaproteobacteria* taxa, *Balneatrix*, SAR86 clade and ZD0405 clade (**Figure 6**) showed only minor representation in sea ice, and were absent from the deep-sea samples. The most dominant *Gammaproteobacteria* in the sediment were members of the JTB255 marine benthic group, and these were restricted to deep-sea samples. Deep-sea surface sediment additionally showed a strong contribution of *Deltaproteobacteria* and *Acidimicrobiia* (**Figure 5b**).

*Alphaproteobacteria* contributed most to the communities in surface seawater, deep-sea sediment and the digestive tract content of holothurians, while their sequence abundance in sea ice and melt ponds was low (**Figure 5b**). While the genus *Planktomarina* and members of the SAR11 Surface1 clade predominated in the water column, *Rhodospirillaceae* were the most dominant representatives at the seafloor. The bacterial community in the holothurian digestive tract was very different from any other environment (**Figure 5b**) and almost entirely comprised of the alphaproteobacterial genus *Sedimentitalea* and the genus *Rubritalea* from the *Verrucomicrobiae* (**Figure 6**).

Based on OTU presence/absence, the microbial community associated with sea-ice algae aggregates showed highest community overlap with both melt-pond water and sea-ice communities. We found lower similarity in composition with the underlying community in surface seawater (**Figure 3**). Interestingly, the community overlap between ice-algal aggregates associated with melt ponds and the exported ice algae at the seafloor was comparable to or higher than the overlap between aggregates and surface seawater for both bacteria and eukaryotes (**Figure 3**), despite the >4000 m descent. At the seafloor, deposited ice-algal aggregate communities exhibited similar proportions of community overlap with the surrounding sediment as with the sea-ice bottom and surface seawater. A large overlap was also observed between eukaryotic communities in the deposited ice algae and the digestive tract content of the deep-sea dwelling holothurians (**Figure 3b**). Sea ice and sediment, as well as surface seawater and sediment communities showed lowest community overlap.

Only three bacterial and seven eukaryotic OTUs in the datasets fit our definition of generalists present across the Arctic habitats. These included members of the genera *Colwellia*, *Oleispira* (both *Gammaproteobacteria*) and *Lentimonas* (*Opitutae*) (Supplementary Information Discussion). Eukaryotic generalists included members of the genera *Cryothecomonas* (*Thecofilosea*), the NIF-3A7 and NW617 clades (*Thecofilosea*) and unclassified *Dinophyceae*, but also the photosynthetic *Micromonas* (*Mamiellophyceae*) and unclassified *Prasinophytae*. We identified a larger number of OTUs in the bacterial and eukaryotic dataset that were shared members between sea-ice algae aggregates in melt ponds, sea ice, water column and deposited algae at the seafloor, but that could not be identified as abundant and dispersed members of the sediment community (Supplementary Tables S3, S4). Overall, a large fraction of community members were also associated with unclassified groups with no closely related cultured representatives, e.g., unclassified *Suessiaceae*, unclassified *Flavobacteriaceae*, SAR86 clade and ZD0405 clade, the JTB255 marine benthic group; their ecological role thus remains elusive.

### Microbial Community Composition Associated With Sea-Ice Algal Aggregates

Visual inspection by light microscopy showed a predominance of *Melosira* in all sampled aggregates retrieved from the sea ice (Fernández-Méndez et al., 2014). Other diatoms observed under the microscope belonged to the pennate genera *Nitzschia*, *Fragilariopsis*, and *Cylindrotheca* (Supplementary Table S1). The ubiquitous presence of the genus *Melosira* (*Coscinodiscophyceae*) and of the above diatoms, as well as golden-brown algae (*Chrysophyceae*) was also revealed by sequencing (Supplementary Table S5). *Melosira* 16S rRNA gene contribution was <0.01% in the degraded aggregates of station Ice1 composed of empty *Melosira* frustrules (Supplementary Table S5), but dominated sequences in the fresh, green aggregate of station Ice7 (**Figure 6**).

Predominant bacterial classes associated with sea-ice algal aggregates were *Flavobacteriia* and *Gammaproteobacteria* (**Figure 5b**). The green aggregate from Ice7 included *Glaciecola* and *Paraglaciecola* (*Gammaproteobacteria*), and *Winogradskyella* (*Flavobacteriia*) (**Figure 6**). The genus *Flavobacterium* (*Flavobacteriia*) was the most abundant genus in the highly degraded aggregates from Ice1, where it contributed up to 89% to the total bacterial sequences. *Flavobacterium* was barely detectable (<0.05%) in the much fresher *Melosira* aggregate from Ice7 (Supplementary Table S6).

Visual inspection of algal aggregates deposited at the seafloor also identified *Melosira* as the dominant component (Boetius et al., 2013; Fernández-Méndez et al., 2014). The eukaryotic sequences retrieved from the deposits fell into two groups: those dominated by diatom sequences, mainly *Melosira*, and unclassified alveolates, and others primarily composed of *Labyrinthulomycetes*, a group of stramenopile protists (**Figure 6**). The eukaryotic community in the holothurian digestive tract resembled the sequence composition of deposited algal aggregates, with a high contribution of the diatom *Melosira* and unclassified alveolates, but also a high contribution of *Cryothecomonas* and *Scrippsiella* observed in sea ice (**Figure 6** and Supplementary Table S5).

*Alpha*-, *Gammaproteobacteria*, and *Flavobacteriia* dominated all deposited algal aggregates (**Figure 5b**), and the overlap with the sea-ice aggregates was 22% at the OTU level. Yet, deposit-associated *Alphaproteobacteria* were dominated by the genera *Octadecabacter*, *Sedimentitalea* and *Sulfitobacter*, which were also detected in sea-ice algae aggregates, but in much lower relative abundances (**Figure 6** and Supplementary Table S6). The relative contribution of *Gammaproteobacteria* was similar in sea-ice algal aggregates and the deposited aggregates at the seafloor, but the predominant genera observed in the deposits, *Neptunomonas*, *Moritella*, and *Colwellia*, differed from the sea-ice aggregates. In contrast, *Winogradskyella*, the most prominent flavobacterial member associated with the deposits was also well represented in sea-ice aggregates. Overall, the contribution of *Flavobacteriia* associated with the deposited aggregates was lower than in the sea-ice aggregates. Instead, the contribution of other classes was higher, including *Epsilonproteobacteria* and *Verrucomicrobiae* (**Figure 5b**). *Epsilonproteobacteria* were entirely represented by the genus *Arcobacter*, which was not detected in any other environment, except for the surrounding surface sediments. *Verrucomicrobiae* were represented by the genus *Rubritalea*, which was also present in sea-ice algae aggregates. The two types of algae deposits that were differentiated by their eukaryotic community composition showed only minor differences in bacterial community composition. *Melosira*-dominated samples showed higher contributions of the genera *Octadecabacter* (on average by a factor of 30), *Sulfitobacter* (on average by a factor of 45), *Ulvibacter* (on average by a factor of 18) and *Winogradskyella* (on average by a factor of 21). Labyrinthulomycetes-dominated samples contained a higher proportion of *Neptunomonas* (on average by a factor of 12; **Figure 6**).

## Discussion

The Arctic Ocean sea-ice cover of the past 10 years shows minima both in extent as well as in thickness relative to the 1981–2010 mean^2^. In autumn 2012, at the time of this study, Arctic sea-ice extent reached a record minimum, a loss that has been unprecedented since the beginning of satellite records. The bottom of the relatively thin ice floes had been populated by diatoms, foremost the colonial *Melosira arctica* forming large filamentous aggregates. Strong melting resulted in a wide-spread export and rapid deposition of sea-ice algal aggregates at the deep-sea floor at around 4,000 m water depth (Boetius et al., 2013). Only few types of benthic fauna, primarily holothurians, were observed to feed on the exported algal material, but a substantial bacterial degradation of the algal deposits was recorded, leading to locally enhanced respiration rates and a depletion of oxygen in and under the algal food falls (Boetius et al., 2013). Other studies have confirmed recent high under-ice productivity and the high contribution of sea-ice algae to export fluxes (Comeau et al., 2013; Lalande et al., 2014; Poulin et al., 2014). Here we compared bacterial and eukaryote community composition on ice-algae aggregates and the surrounding habitats, to understand if the ice-algal aggregates would associate with specific kinds of bacterial groups, if these would be selected from sea ice rather than surrounding waters, and if these communities would get exported to the seafloor. Our analyses did not target archaea, and to our knowledge no data on archaeal community composition is currently available for the Eurasian basin, despite their presence in other Arctic regions (Galand et al., 2009; Comeau et al., 2011), and their potential importance in the biogeochemical cycling of nutrients and carbon (Kirchman et al., 2007). Archaea do not seem to be as tightly linked to phytoplankton biomass, as has been suggested for bacteria (Herfort et al., 2007; Kirchman et al., 2007; Comeau et al., 2011; Wilson et al., 2017), however, their ability to utilize algae-derived organic matter has been demonstrated (Alderkamp et al., 2006; Orsi et al., 2016). As Arctic archaeal communities appear to exhibit high spatio-temporal variability in composition, relative abundance, and heterotrophic activity (Bano et al., 2004; Kirchman et al., 2007; Alonso-Sáez et al., 2008; Galand et al., 2008, 2009; Wilson et al., 2017), future studies of the central Arctic should aim to include archaea in microbial community assessments.

### Ice-Algal Aggregates Are Populated by Sea-Ice Bacteria

Sea-ice algal aggregates were mostly composed of healthy, green or degraded cells of the diatom *Melosira arctica*, but also included a variety of other sea-ice diatoms (Boetius et al., 2013). Microscopic analyses and sequence distribution of eukaryotes largely overlapped. Both eukaryotic and bacterial aggregate sequences showed highest community overlap with the surrounding melt-pond water and the sea ice (**Figure 3**), and the dominant members were also predominant members of the sea-ice and melt-water communities (**Figure 5**). The algal aggregates selected specifically for *Flavobacteriia*, particularly the genus *Flavobacterium*, as well as the gammaproteobacterial *Glaciecola* and *Paraglaciecola* (**Figure 6**). Both are known to be tightly coupled to phytoplankton bloom dynamics (Teeling et al., 2012), and especially members of the *Flavobacteriia* exhibit the ability to hydrolyze complex polymers, such as polysaccharides found in plant and algal cell walls (Humphry et al., 2001; Knoll et al., 2001; Williams et al., 2013). Also for *Glaciecola* a key role in the breakdown of organic matter was suggested, and a specialization on diatom-derived material was observed (Beier et al., 2015). Most members of this genus seem to be psychrophilic, and recent experimental work suggests that cold-adapted *Glaciecola* can be the dominant consumers of algae material in low-temperature environments (von Scheibner et al., 2017). However, bacteria and eukaryotes associated with the ice-algal aggregates both exhibited high beta-diversity, reflecting substantial variations in community structure between individual aggregates. It has been observed before that differently composed phytoplankton blooms may lead to the establishment of distinct bacterial assemblages, due to differences in organic material released by individual algal taxa (Pinhassi et al., 2004; Grossart et al., 2005).

Previous studies observed a dominance of *Betaproteobacteria* in bacterial melt-pond communities (Brinkmeyer et al., 2004), while we detected a clear predominance of *Flavobacteriia* (**Figure 5b**). *Betaproteobacteria* are known as particle colonizers and were shown to play key roles in the decomposition of aggregates and nutrient cycling, especially in freshwater ecosystems (Knoll et al., 2001; Schweitzer et al., 2001; Lemarchand et al., 2006). However, while *Betaproteobacteria* were described as early colonizers of particles, *Flavobacteriia* contribution was shown to increase in the late phase of blooms (Knoll et al., 2001), when they potentially benefit from their capacity to break down refractory material. The high contribution of *Flavobacteriia* in the melt ponds and sea ice may therefore reflect a late or post bloom state of the system, indicated by the low nutrient inventories during the time of the sampling (Fernández-Méndez et al., 2015). Melt ponds, even when closed, can be directly connected to the surface ocean through a network of brine channels that penetrate the sea-ice matrix, allowing exchange of communities between environments (Boetius et al., 2015). However, the community in melt-pond water differed considerably from that of seawater, even at ice station 7, where the melt pond was open and salinity in the pond was comparable to the underlying water column (**Figure 6**). Our results, therefore, underpin a recruitment of bacterial groups mainly from sea ice, best adapted to the utilization of ice algal material in the ponds, which differed from the phytoplankton material in the surface ocean. We observed first indications for specific associations between bacterial groups and ice-algae aggregates, with 12 dispersed and abundant bacterial OTUs as exclusive members of ice-algae aggregates and algae deposits (Supplementary Table S3). These comprised mainly *Alphaproteobacteria*, including several members of the Roseobacter clade, e.g., *Sulfitobacter*. Examples of both mutualistic and pathogenic interactions have been described for Roseobacter-algae associations, with important implications especially for carbon and sulfur cycling in the environment (Ramanan et al., 2016). Roseobacter are known as rapid colonizers of algae surfaces, where they often outcompete other bacterial groups, probably facilitated by their ability to sense and utilize several compounds released by phytoplankton, including dimethylsulfoniopropionate (Buchan et al., 2014).

### Ice-Algal Aggregates Transport Sea-Ice Microorganisms to the Deep-Sea Floor

Comparative analyses of microbial communities from upper ocean and deep sea showed that these communities barely overlap (Countway et al., 2007; Amaral-Zettler et al., 2010; Zinger et al., 2011; Walsh et al., 2016). Recent studies targeting the local overlap between surface water and surface sediment communities indicated a role of sinking particles and overlying water column properties in structuring benthic bacterial communities (Nagata et al., 2000; Hamdan et al., 2013; Ruff et al., 2014; Lindh et al., 2017). Here we investigated whether the rapidly sinking sea-ice algae deliver their associated bacteria to the deep-sea floor. *Melosira* filaments differ from other sinking particles in their large size and concomitant fast sinking rate (Alldredge and Gotschalk, 1988; Fernández-Méndez et al., 2014), with aggregates of a diameter of 3 cm potentially reaching the deep-sea floor within a single day after losing buoyancy at the surface (Katlein et al., 2014). We observed algae deposits, which were almost entirely composed of fresh *Melosira* and alveolates from the sea ice, with intact chloroplasts, presenting a fresh carbon source for benthic communities. These aggregates can potentially transport large numbers of microbial cells from the surface to the seafloor. In the most degraded *Melosira* aggregates, the dominant sequences belonged to members of the *Labyrinthulomycetes* (**Figure 6**), a group of heterotrophic protists that play important roles as saprobes of dead algal material and marine snow (Raghukumar, 2002; Bochdansky et al., 2017). *Labyrinthulomycetes* display an absorptive mode of nutrition and possess the ability to chemically alter and degrade algal detritus through the production of extracellular enzymes (Bahnweg, 1979; Raghukumar, 2002). Interestingly, in the investigated holothurian digestive tract *Melosira* sequences dominated largely over those of *Labyrinthulomycetes*, indicating a preference of the holothurians for fresh deposits (**Figure 6**).

We detected a large proportion of shared bacterial OTUs (22%) belonging to both the abundant types present on sea-ice algae aggregates and on deposited aggregates at the seafloor (**Figure 3a** and Supplementary Table S3). They included representatives of the genera *Paraglaciecola*, *Glaciecola* (both *Gammaproteobacteria*), *Octadecabacter* (*Alphaproteobacteria*), *Psychroserpens* and *Polaribacter* (*Flavobacteriia*), which have been previously described as psychrophiles, and were observed in sea ice and surface seawater (Bowman et al., 1997, 2012; Gosink et al., 1997; Junge et al., 2002; Brinkmeyer et al., 2003; Han et al., 2014; Hatam et al., 2014). While these OTUs represented 37% of the bacterial sequences associated to sea-ice aggregates and were also abundant members in sea ice, they contributed only 1% of the sequences in the deposits and were absent or represented by a minor fraction in sediments (Rapp et al., 2017). We therefore conclude that they were exported with the aggregates, but overgrown by other bacteria.

### Specific Sediment Microbes Overgrow Deposited Algal Aggregates

A considerable amount of bacteria can be exported to the deep sea attached to particles (Turley and Mackie, 1995), but their fate and biogeochemical roles remain elusive. Indigenous deep-sea bacteria are likely better adapted to *in situ* temperature and pressure of the deep-sea environment than surface-derived bacteria (Turley and Lochte, 1990; Turkey, 1993; Poremba, 1994; Tamburini et al., 2006, 2013). The results of this study suggest that the deposited algal aggregates were overgrown within a few weeks to months by specific bacterial groups of the surrounding sediment (**Figure 6**). Accordingly, the bacterial genera of highest relative sequence abundance (i.e., up to 37%) in the deposited ice-algae aggregates consisted of benthic groups that were absent from ice-associated environments and only of minor relative abundance in the surrounding sediment surface (**Figure 6** and Supplementary Table S6). Most prominent were members of the alphaproteobacterial Roseobacter clade, i.e., the genus *Sedimentitalea*, but also the genera *Arcobacter* (*Epsilonproteobacteria*) and *Neptunomonas* (*Gammaproteobacteria*) (**Figure 6**). The Roseobacter clade is a physiologically versatile group, known for its capability to utilize a wide range of organic and inorganic compounds and its tight interactions with phytoplankton, important for organic matter decomposition (Buchan et al., 2014 and references therein). *Arcobacter* has been recorded in a variety of sediment environments, including surface sediment from the Antarctic shelf (Bowman and McCuaig, 2003), the Wadden Sea (Llobet-Brossa et al., 1998) and the deep sea (Thamdrup et al., 2000). Its capacity to attach to surfaces (Assanta et al., 2002), denitrify (Heylen et al., 2006), fix nitrogen (Wirsen et al., 2002), as well as its ability to perform dissimilatory manganese reduction (Thamdrup et al., 2000) and recycle sulfur (Wirsen et al., 2002) indicate a potentially important role of *Arcobacter* in re-mineralizing nutrients from aggregates. Suboxic patches occurred under the algal aggregates in otherwise fully oxygenated sediments (Boetius et al., 2013), potentially selecting for *Arcobacter*. Members of the *Neptunomonas* genus have been isolated from sediments (Zhang et al., 2010), but were also found associated to unicellular eukaryotes (Frommlet et al., 2015) or in close vicinity to whale carcasses (Miyazaki et al., 2008). This genus is known for its capacity to degrade polycyclic aromatic hydrocarbons (Hedlund et al., 1999), indicating an involvement in the breakdown of recalcitrant carbon sources in the deep sea. The selection of rare sediment taxa best adapted to the utilization of the deposited algal detritus could on the long term induce shifts in the indigenous benthic bacterial communities, as has recently been observed in deep-sea surface sediments along the Antarctic Polar Front (Ruff et al., 2014). Overall, the algal deposits changed community composition locally at the deep-sea floor not only by introducing surface ice-bacteria, but also by selecting for specific sediment community members, which resulted in a unique community profile, distinct from any source community (**Figures 4a**, **6**). It remains to be further investigated whether this is a transient feature or whether such events, if reoccurring, will result in a gradual shift of deep-sea sediment communities.

As areas of strong summer ice melt are expanding in the central Arctic, it is expected that abrupt export events may become more frequent in areas covered by seasonal sea ice. This is supported by observations during recent expeditions in late summer 2016, where large areas of the central Arctic seafloor were again covered with *Melosira* deposits (Soltwedel, 2016; Boetius and Purser, 2017). Consequently, recurrent deposition of sea ice algae at the seafloor could facilitate the establishment of unique microbial assemblages as has been proposed for other discrete resource patches (Grassle and Morse-Porteous, 1987; Durán et al., 2013; Lindh et al., 2017) and would then likely lead to changes in bacterial community structure and in the biogeochemical cycling of carbon and nutrients in surface sediments.

### Connectivity of Microbial Communities in the Central Arctic Ocean and Potential Effects of Climate Change

Bacteria in polar ice-associated environments need to be well-equipped to quickly adapt to changes and cope with rapid transitions of seasonal extremes, ranging from high salt concentration in brine inclusions to freshwater salinity in melt ponds (Deming, 2010), and protect themselves against sub-zero temperatures. Several studies showed the production of so-called compatible solutes by some sea-ice bacteria, which they can use as osmo- and cryoprotectants (Methe et al., 2005; Bowman, 2008). These specific adaptations to life around the freezing point of water may also facilitate the exchange of bacteria between habitats, e.g., after ice melt and export of attached bacteria in aggregates. We detected a high number of abundant OTUs shared between sea ice, melt ponds and aggregates, but only a low number of generalist OTUs (Supplementary Information Discussion and Supplementary Table S5), i.e., OTUs present in all sampled environments, despite the relatively uniform temperatures below zero degree.

Hence, this study confirms that sea-ice bacterial and eukaryotic communities are distinct from other Arctic habitats such as seawater and sediment (**Figure 4**). Consequently, ice dwelling organisms, and especially multi-year ice communities, which are distinct from those of seasonal ice (Leu et al., 2011; Bowman et al., 2012; Boetius et al., 2015; Hatam et al., 2016), may be lost from an Arctic devoid of summer sea ice, with unknown repercussions for productivity, organic matter cycling and other ecological functions. In addition, ice melt may contribute to upper water column freshening, potentially inducing shifts in the bacterioplankton composition, as has been observed for the phytoplankton community (Li et al., 2009). We saw first indications for a bacterial surface seawater community response to massive ice melt in the predominance of the gammaproteobacterial genus *Balneatrix*, which was previously described to thrive under freshwater conditions (González and Whitman, 2006), and members of the genus *Planktomarina* of the Roseobacter clade (**Figure 6**), which was observed to correlate negatively with salinity (Giebel et al., 2011). Other than previous studies on bacterial community composition in the Arctic water column, we did not observe a predominance of the SAR11 clade (Kirchman et al., 2010; Bowman et al., 2012). This may be due to a primer bias (Apprill et al., 2015; Parada et al., 2016), but may also be ascribed to a lower representation of this oligotrophic clade during the post-bloom state of the system at the time of sampling. We observed a significant correlation between bacterial and eukaryote community patterns (Supplementary Figure S7), likely indicating similar drivers of diversification and potentially also biotic interactions, which remain largely unknown for polar organisms to date (Lima-Mendez et al., 2015). Future efforts should therefore aim to integrate bacterial, archaeal and eukaryotic community analyses, and expand the temporal resolution of sampling to better resolve seasonal dynamics.

## Author Contributions

JR and MF-M designed the study, with contributions by CB and AB. MF-M, CB, and AB collected the samples. JR performed the laboratory molecular and bioinformatic analysis. JR and CB performed the statistical analysis. JR interpreted the data and all authors contributed to the discussion of the results. JR wrote the manuscript, with support and input from all co-authors.

## Conflict of Interest Statement

The authors declare that the research was conducted in the absence of any commercial or financial relationships that could be construed as a potential conflict of interest.

## Footnotes

1 https://sourceforge.net/projects/bbmap

2 http://nsidc.org
