# Supplementary material for: Effects of Ice-Algal Aggregate Export on the Connectivity of Bacterial Communities in the Central Arctic Ocean
Source: Front Microbiol. 2018 May 18;9:1035. doi: 10.3389/fmicb.2018.01035 (PMC5974969; doi:10.3389/fmicb.2018.01035)
Supplement: Supplementary file 3 [file Data_Sheet_2.DOCX]

# Analysis of 18S amplicons

# Based on a script adpated from the OSD 16S pipeline (https://colab.mpi-bremen.de/micro-b3/svn/analysis-scripts/trunk/osd-analysis/osd-pre-processing/16S/lgc/primer-clipping/02primer-clipping.sh author: Antonio Fernandez-Guerra) by Christiane Hassenrueck at the MPI Bremen. Swarming and (some of) the classification (additional credits: Chris Quast, Josephine Rapp, Pier Buttigieg) differs from the OSD pipeline.

# reads generated on a MiSeq, 2x300bp

# customized for sequencing at CeBiTec in Bielefeld (Halina Tegetmeyer)

# input seqeunces do NOT contain barcode and adapter sequences anymore, but still contain primer sequences

# to avoid weird line endings (^M) work exclusively on linux when preparing and creating files (or make sure you convert to Linux line endings)

# workflow optimzed for parallelization on aphros (PBS) at the AWI

# more R scripts can be found at www.github.com/chassenr/NGS in the AMPLICON and Plotting directory

#######################################

#

# Preliminaries...

#

#######################################

# Directories...

# This workflow is started in the directory ${FILELOCATION} which contains all raw sequence (fastq) files.

# Any change of directory later on in the workflow is included in the script

# set variables, program and script locations

FILELOCATION="XX" #starting directory, location of raw sequence files

NSAMPLE="XX" #number of samples

SINA_PT="XX/db/SILVA/SSURef_NR99_123_SILVA_12_07_15_opt.arb" #database used for taxonomic classification

#######################################

#

# General steps...

#

#######################################

#step 1: primer clipping (cutadapt, parameters from OSD workflow)

#step 2: quality trimming (trimmomatic) - for long reads recommended after merging: per base quality might improve throughout merging progress

#step 3: merging (PEAR)

#step 4: quality trimming and control (trimmomatic, fastqc)

#step 5: otu clustering using swarm

#step 6: taxonomic classification of seed sequences

#step 7: further analysis (R)

######################################

#

# Analysis pipeline

#

######################################

###step 1: primer clipping

# (adapted from https://colab.mpi-bremen.de/micro-b3/svn/analysis-scripts/trunk/osd-analysis/osd-pre-processing/16S/lgc/primer-clipping/02primer-clipping.sh)

# for each sample the following commands will remove the primer sequence when found (or discard the read)

# it will search both R1 and R2 for both the forward and the reverse primer sequence and

# if the insert is inverted, it will turn the sequences in the right orientation to make life easier downstream...

# Input your primer sequences, use ^ to anchor to the beginning of line

# TAReukFWD1-TAReukREV3 V4 region (~380 bp)

#TAReukFWD1 (5′-CCAGCASCYGCGGTAATTCC-3′)

#TAReukREV3 (5′-ACTTTCGTTCTTGATYRA-3′)

FBC=^CCAGCASCYGCGGTAATTCC # forward primer

RBC=^ACTTTCGTTCTTGATYRA # reverse primer

OFWD=19 # length of forward primer (20) - 1

OREV=17 # length of reverse primer (18) - 1

# Set the proportion of mismatches allowed when matching the primer sequences

ERROR=0.16

# create directory for output of primer clipping

mkdir Clipped

# performed as an array job via the queuing system

# primer clipping

# Let's process the reads in the FR orientation...

## Look for forward primer in R1. R2 is processed at the same time to make sure these files go

## through the same processes and any sequences discarded match between outputs.

## If primer is not found and no trimming is done, both R1 and R2 are discarded

## temporary, log, and information files are placed in the sample directory...

cutadapt --no-indels -O ${OFWD} -g ${FBC} -e ${ERROR} --info-file ./Clipped/${PBS_ARRAYID}"_clip_fr.R1.info" -o ./Clipped/${PBS_ARRAYID}"_clip_R1.TMP.fastq" -p ./Clipped/${PBS_ARRAYID}"_clip_R2.TMP.fastq" ./Renamed/${PBS_ARRAYID}"_R1.fastq" ./Renamed/${PBS_ARRAYID}"_R2.fastq" --untrimmed-o /dev/null --untrimmed-p /dev/null > ./Clipped/${PBS_ARRAYID}"_clip_fr.R1.cutadapt.log" 2>&1

## Using the output of the previous command (the TMP files), we'll now look for the reverse primer in R2, bringing R1 along for the ride.

## note inverse orientation of input files. As before, if primer is not found, remove the pair.

## this step generates the final results for the FWD-REV orienatation, accepting those seqs that passed the first stage.

cutadapt --no-indels -O ${OREV} -g ${RBC} -e ${ERROR} --info-file ./Clipped/${PBS_ARRAYID}"_clip_fr.R2.info" -o ./Clipped/${PBS_ARRAYID}"_clip_R2.fastq" -p ./Clipped/${PBS_ARRAYID}"_clip_R1.fastq" ./Clipped/${PBS_ARRAYID}"_clip_R2.TMP.fastq" ./Clipped/${PBS_ARRAYID}"_clip_R1.TMP.fastq" --untrimmed-o /dev/null --untrimmed-p /dev/null > ./Clipped/${PBS_ARRAYID}"_clip_fr.R2.cutadapt.log" 2>&1

## clean up TMP files...

rm ./Clipped/${PBS_ARRAYID}"_clip_R1.TMP.fastq"

rm ./Clipped/${PBS_ARRAYID}"_clip_R2.TMP.fastq"

# Now to process the reads with the RF orientation...

## First, we search for the reverse primer in R1...

cutadapt --no-indels -O ${OREV} -g ${RBC} -e ${ERROR} --info-file ./Clipped/${PBS_ARRAYID}"_clip_rf.R1.info" -o ./Clipped/${PBS_ARRAYID}"_clip_R1.TMP.fastq" -p ./Clipped/${PBS_ARRAYID}"_clip_R2.TMP.fastq" ./Renamed/${PBS_ARRAYID}"_R1.fastq" ./Renamed/${PBS_ARRAYID}"_R2.fastq" --untrimmed-o /dev/null --untrimmed-p /dev/null > ./Clipped/${PBS_ARRAYID}"_clip_rf.R1.cutadapt.log" 2>&1

## As before, we search for the forward primer in R2, only processing the output of the previous command

cutadapt --no-indels -O ${OFWD} -g ${FBC} -e ${ERROR} --info-file ./Clipped/${PBS_ARRAYID}"_clip_rf.R2.info" -o ./Clipped/${PBS_ARRAYID}"_clip_rf.R2.fastq" -p ./Clipped/${PBS_ARRAYID}"_clip_rf.R1.fastq" ./Clipped/${PBS_ARRAYID}"_clip_R2.TMP.fastq" ./Clipped/${PBS_ARRAYID}"_clip_R1.TMP.fastq" --untrimmed-o /dev/null --untrimmed-p /dev/null > ./Clipped/${PBS_ARRAYID}"_clip_rf.R2.cutadapt.log" 2>&1

## remove the temp files, as -o ${RFR2} -p ${RFR1} have this step's results

rm ./Clipped/${PBS_ARRAYID}"_clip_R1.TMP.fastq"

rm ./Clipped/${PBS_ARRAYID}"_clip_R2.TMP.fastq"

# Reorient REV-FWD output to FWD-REV

# Change the read id in the headers "@MISEQ:41:000000000-A9A9U:1:1101:17488:1966 1:N:0:AGGCAGAAAGAGTAGA"

awk '{if (NR%4==1){gsub("^1:","2:",$2); print $0}else{print $0}}' ./Clipped/${PBS_ARRAYID}"_clip_rf.R1.fastq" > ./Clipped/${PBS_ARRAYID}"_rf2frR2.fastq"

awk '{if (NR%4==1){gsub("^2:","1:",$2); print $0}else{print $0}}' ./Clipped/${PBS_ARRAYID}"_clip_rf.R2.fastq" > ./Clipped/${PBS_ARRAYID}"_rf2frR1.fastq"

# rename the reorient. rev-fwd files

mv ./Clipped/${PBS_ARRAYID}"_rf2frR1.fastq" ./Clipped/${PBS_ARRAYID}"_clip_rf.R1.fastq"

mv ./Clipped/${PBS_ARRAYID}"_rf2frR2.fastq" ./Clipped/${PBS_ARRAYID}"_clip_rf.R2.fastq"

# add all corrected seqs to the fwd-rev fastqs

cat ./Clipped/${PBS_ARRAYID}"_clip_rf.R1.fastq" >> ./Clipped/${PBS_ARRAYID}"_clip_R1.fastq"

cat ./Clipped/${PBS_ARRAYID}"_clip_rf.R2.fastq" >> ./Clipped/${PBS_ARRAYID}"_clip_R2.fastq"

# cleaning up directories

mkdir ./Clipped/Clipped_logs

mv ./Clipped/*.log ./Clipped/Clipped_logs/

mv ./Clipped/*.info ./Clipped/Clipped_logs/

###step 2: quality trimming

# for long inserts (450ish and onwards) recommended after merging

# (you need all the bases you can get, additionally two identical bases with low quality which are merged will generally have a higher quality score.

# shorter inserts have more overlap and can afford some loss)

# ptrim = identical sequence headers with R1 and R2

# strim = single output, complementary reads removed

# SLIDINGWINDOW:4:15

# argument order matters! MINLEN should come after trimming.

# for each sample, trim the clipped reads with a sliding window of 4 and a quality threshold of 15

# Discard reads less than 100 bps.

# creating directory for output of quality trimming

mkdir Trimmed

# performed as an array job via the queuing system

java -jar /opt/bio/Trimmomatic-0.35/trimmomatic-0.35.jar PE -threads 2 -trimlog ./Trimmed/${PBS_ARRAYID}"_trim.log" ./Clipped/${PBS_ARRAYID}"_clip_R1.fastq" ./Clipped/${PBS_ARRAYID}"_clip_R2.fastq" ./Trimmed/${PBS_ARRAYID}"_ptrim_R1.fastq" ./Trimmed/${PBS_ARRAYID}"_strim_R1.fastq" ./Trimmed/${PBS_ARRAYID}"_ptrim_R2.fastq" ./Trimmed/${PBS_ARRAYID}"_strim_R2.fastq" SLIDINGWINDOW:4:15 MINLEN:100

# cleaning up directories

mkdir ./Trimmed/Trimmed_logs

mv ./Trimmed/*.log ./Trimmed/Trimmed_logs

###step 3: read merging (CHECK RESOURCES!!!)

# this will merge reads with a minimum overlap of 30 (-v)

# the minimum length of the merged reads is 350 (-n)

# for short insert sizes it might be recommented to set a maximum length for the merged reads (here -m 500).

# Freakishly long reads generally indicate an error...

# j: threads

# v: overlap

# n: min insert length

# m: max insert length

# o: output just needs basename, other stuff is added by PEAR

# no trimming (q) enabled as trimmomatic did the work here.

#creating directory for output of merging

mkdir Merged

# performed as an array job via the queuing system

pear -j 2 -v 30 -n 350 -m 500 -f ./Trimmed/${PBS_ARRAYID}"_ptrim_R1.fastq" -r ./Trimmed/${PBS_ARRAYID}"_ptrim_R2.fastq" -o ./Merged/${PBS_ARRAYID} > ./Merged/${PBS_ARRAYID}"_merged.log"

# cleaning up directories

mkdir ./Merged/Merged_logs

mv ./Merged/*.log ./Merged/Merged_logs

###step 4: quality control with FASTQC

# create directory for fastqc output

mkdir FastQC

# performed as an array job via the queuing system

cd ./FastQC/

time fastqc -o . ../Merged/${PBS_ARRAYID}".assembled.fastq"

unzip ${PBS_ARRAYID}".assembled_fastqc.zip"

cd ..

# output some diagnostic files

# combine flags of 'Per base sequence quality' module for all files

grep "Per base sequence quality" ./FastQC/[0-9]*.assembled_fastqc/summary.txt > ./FastQC/QC_summary.txt

# range of read lengths

grep "Sequence length" ./FastQC/[0-9]*.assembled_fastqc/fastqc_data.txt > ./FastQC/QC_read_length.txt

# combine flags of 'Sequence Length Distribution' module for all files including most abundant read lengths

for i in $(seq 1 ${NSAMPLE})

do

awk '/^>>Sequence Length Distribution/,/^>>END_MODULE/' ./FastQC/$i".assembled_fastqc"/fastqc_data.txt |\

sed -e '1,2d' -e '$d' > ./FastQC/$i".assembled_fastqc"/fastqc_SLD.txt

sort -t$'\t' -k2nr ./FastQC/$i".assembled_fastqc"/fastqc_SLD.txt |\

head -1 |\

paste <(grep "Sequence Length Distribution" ./FastQC/$i".assembled_fastqc"/summary.txt) -

done > ./FastQC/QC_read_distribution.txt

# count sequences

# only counting forward read as representative for PE

grep -c '^@MISEQ' ./Renamed/[0-9]*_R1.fastq > nSeqs_all.txt

grep -c '^@MISEQ' ./Clipped/[0-9]*_clip_R1.fastq >> nSeqs_all.txt

grep -c '^@MISEQ' ./Trimmed/[0-9]*_ptrim_R1.fastq >> nSeqs_all.txt

grep -c '^@MISEQ' ./Merged/[0-9]*.assembled.fastq >> nSeqs_all.txt

###step 5: swarm OTU clustering (https://github.com/torognes/swarm)

# create directory for swarm input and output

mkdir Swarm

# extract fasta file from fastq and move to new directory

# requires at least jre1.8

# set fastawrap to 1000 to prevent line breaks within sequence

reformat.sh in=./Merged/${PBS_ARRAYID}".assembled.fastq" out=./Swarm/${PBS_ARRAYID}"_good.fasta" fastawrap=1000

# Now to dereplicate and rename individual reads to save compute and mental anguish downstream...

# The dereplication code is courtesy of the Swarm developers and can be found here:

# https://github.com/torognes/swarm/wiki/Working-with-several-samples

cd ./Swarm/

grep -v "^>" ${PBS_ARRAYID}"_good.fasta" | \

grep -v [^ACGTacgt] | sort -d | uniq -c | \

while read abundance sequence ; do

hash=$(printf "${sequence}" | sha1sum)

hash=${hash:0:40}

printf ">%s_%d_%s\n" "${hash}" "${abundance}" "${sequence}"

done | sort -t "_" -k2,2nr -k1.2,1d | \

sed -e 's/\_/\n/2' > ${PBS_ARRAYID}"_dereplicated.fasta"

cd ..

# study level dereplication

cd ./Swarm/

export LC_ALL=C

cat *_dereplicated.fasta | \

awk 'BEGIN {RS = ">" ; FS = "[_\n]"}

{if (NR != 1) {abundances[$1] += $2 ; sequences[$1] = $3}}

END {for (amplicon in sequences) {

print ">" amplicon "_" abundances[amplicon] "_" sequences[amplicon]}}' | \

sort --temporary-directory=$(pwd) -t "_" -k2,2nr -k1.2,1d | \

sed -e 's/\_/\n/2' > all_samples.fasta

#building amplicon contingency table (use script from swarm 1.20)

#the python script supplied with the latest versions of swarm may not work properly

python amplicon_contingency_table.py *_dereplicated.fasta > amplicons_table.csv

#swarming

# -b light swarms have less than 3 reads associated with them

# -d 1: local edit distance threshold is 1

# fastidious algorithm (-f): light swarms (amplicon abundance less than 3) will be grafted to heavy swarms

# -t set threads to 4

# -l output a log file

# -o the swarm file itself

# -s output a stats file (needed downstream)

# -w output fasta file with seed sequences

swarm -b 3 -d 1 -f -t 8 -l swarm.log -o amplicons.swarms -s amplicons_stats.txt -w amplicons_seeds.fasta all_samples.fasta

# building OTU contingency table for multiple samples

# https://github.com/torognes/swarm/wiki/Working-with-several-samples

# let the script know where the good stuff is...

STATS="amplicons_stats.txt"

SWARMS="amplicons.swarms"

AMPLICON_TABLE="amplicons_table.csv"

OTU_TABLE="OTU_contingency_table.csv"

# Header

echo -e "OTU\t$(head -n 1 "${AMPLICON_TABLE}")" > "${OTU_TABLE}"

# Compute "per sample abundance" for each OTU

awk -v SWARM="${SWARMS}" -v TABLE="${AMPLICON_TABLE}" 'BEGIN {FS = " "

while ((getline < SWARM) > 0) {

swarms[$1] = $0

}

FS = "\t"

while ((getline < TABLE) > 0) {

table[$1] = $0

}

}

{# Parse the stat file (OTUs sorted by decreasing abundance)

seed = $3 "_" $4

n = split(swarms[seed], OTU, "[ _]")

for (i = 1; i < n; i = i + 2) {

s = split(table[OTU[i]], abundances, "\t")

for (j = 1; j < s; j++) {

samples[j] += abundances[j+1]

}

}

printf "%s\t%s", NR, $3

for (j = 1; j < s; j++) {

printf "\t%s", samples[j]

}

printf "\n"

delete samples

}' "${STATS}" >> "${OTU_TABLE}"

# You may want to check if large swarms are taxonomically consistent

# by classifying more than their seed sequences.

###step 6: taxonomic classification

# At this stage, you could consider removing very rare swarms (less than one or two reads per swarm).

# As a large chunk of the swarms are rare (and will probably be removed from analysis later), you can save compute time here

# As always, whether this is advisable or not depends on your question.

# convert lowercase sequences to uppercase sequences in amplicons_seeds.fasta

awk '{print /^>/ ? $0 : toupper($0)}' amplicons_seeds.fasta > amplicons_seeds_uc.fasta

# removal of singletons

# this will output the accession numbers of all non-singleton swarms

# and a table with the precentage of retained sequences per sample if singleton swarms are removed

Rscript

otu=read.table("OTU_contingency_table.csv",h=T,sep="\t")

otu2=otu[,3:(ncol(otu)-1)]

singletons=colSums(otu2[otu$total>1,])/colSums(otu2)

write.table(singletons,"retainedSequences.txt",quote=F)

write.table(data.frame(as.character(otu[otu$total>1,2])),"heavy.accnos",row.names=F,col.names=F,quote=F)

# rename the original amplicons_seeds_uc.fasta so that it is not overwritten

mv amplicons_seeds_uc.fasta amplicons_seeds_uc_all.fasta

# select only the representative sequences of non-singleton swarms

grep -A1 -F -f heavy.accnos amplicons_seeds_uc_all.fasta | sed '/^--$/d' > amplicons_seeds_uc.fasta

cd ..

# splitting seed sequence fasta file for parallel processing

mkdir Sina

cd Sina

asplit '^>' 2000 < ../Swarm/amplicons_seeds_uc.fasta #split fasta file in fasta files with 2000 sequences each

# Determine how many chunks there are...

JOBCOUNT=$(ls -1 out* | wc -l) #specify the number of files in array job

sina -i out.${PBS_ARRAYID} --intype fasta -o sina_out.${PBS_ARRAYID} --outtype fasta --search --meta-fmt csv --overhang remove --insertion forbid --filter none --fs-kmer-no-fast --fs-kmer-len 10 --fs-req 2 --fs-req-full 1 --fs-min 40 --fs-max 40 --fs-weight 1 --fs-full-len 1400 --fs-msc 0.7 --match-score 1 --mismatch-score -1 --pen-gap 5 --pen-gapext 2 --search-cover query --search-iupac optimistic --search-min-sim 0.9 --turn all --lca-quorum 0.7 --search-db ${SINA_PT} --ptdb ${SINA_PT} --lca-fields tax_slv

# check that all sequences were classified

# no output is good, otherwise the sequence number per 2000-sequence package is printed to standard output

for file in ${FILELOCATION}/Logfiles/sina.e*

do

find_missing_lca.py $file

#file contains:

import sys

sequence = None

foundLCA = False

for line in open(sys.argv[1]) :

if line[0:15] == "sequence_number" :

if sequence and not foundLCA :

print sys.argv[1] + ":" + sequence

foundLCA = False

sequence = line[16:]

elif line[0:11] == "lca_tax_slv" :

foundLCA = True

if sequence and not foundLCA :

print sys.argv[1] + ":" + sequence

done

# Time to gather up the useful info from the split output...

# In grep, -h suppresses printing of filenames for results

# Get all the swarm seed hashes (sort of like accessions)

grep -h '^sequence_identifier' $(ls -1v ${FILELOCATION}/Logfiles/sina.e*) | sed 's/^sequence_identifier: //' > amplicons_seeds.accnos

#check if the order is the same as in amplicons_seeds_uc.fasta

grep '^>' ../Swarm/amplicons_seeds_uc.fasta | sed 's/^>//' | diff - amplicons_seeds.accnos

# Get all corresponding taxonomic paths (note the same order as the accnos)

grep -h '^lca_tax_slv' $(ls -1v ${FILELOCATION}/Logfiles/sina.e*) | sed 's/^lca_tax_slv: //' > amplicons_seeds.tax_slv

# Get all alignment qualities (for filtering later)

grep -h '^align_quality_slv' $(ls -1v ${FILELOCATION}/Logfiles/sina.e*) | sed 's/^align_quality_slv: //' > amplicons_seeds.align_quality_slv

# merge these output files...

paste amplicons_seeds.accnos amplicons_seeds.align_quality_slv amplicons_seeds.tax_slv > amplicons_seeds_taxonomy.txt

cd ..

# copy final output files to working directory

cp ./Swarm/OTU_contingency_table.csv ./

cp ./Sina/amplicons_seeds_taxonomy.txt ./

###step 7: further analysis (R)

# use e.g. ReadAmplicon.R, SubsampleNGS.R, PlotHillNGS.R

# download at www.github.com/chassenr/NGS in AMPLICON and Plotting directory

#transfer OTU_contingency_table.csv, amplicons_seeds_taxonomy.txt, SILVA123_tax_slv_ssu_curated.tsv to local computer for further processing in R

#start RStudio

source("ReadAmplicon.R")

source("SubsampleNGS.R")

# reading output of 16S workflow

# removing unwanted lineages

# parsing taxonomic path according to SILVA (further manual curation may be necessary)

X_Euk <- ReadAmplicon(otu = "OTU_contingency_table.csv", tax = "amplicons_seeds_taxonomy.txt", silva = "SILVA123_tax_slv_ssu_curated.tsv", domain = "Bacteria", silva.sep = "\t", singletons = F, unclassified = T, write.files = T)

#calculating Alpha diversity indices based on n random subsampling runs to e.g. the minimum library size (sub)

#removed samples with read numbers <10,000 from alpha diversity analysis

#subset_X_Euk_10000 <-X_Euk$OTU[,-13] #step by step removal of low read samples (total of 6)

min(colSums(X_Euk$OTU)))

Alpha_Euk <- SubsampleNGS(X_Euk$OTU, n = 100, sub = min(colSums(X_Euk$OTU)))

####calculate shared OTU between samples

##shared between core communities -> overlap presence absence of OTUs that are present as core (present in majority of samples from one environment)

#manually create input OTU table:

#for bacteria: IceS_Core -> if present in >=4; IceB_Core -> if present in >=4; IceBr_Core -> if present in =2; MPW_Core -> if present in >=2; MPAGG_Core -> if present in >=2; SW_Core -> if present in >=4; DSAGG_Core -> if present in >=4; HGC_Core -> if present in =2; Sed_Core -> if present in >=5

taxapooler_in_euk_PA <- read.table("./euk/X_IceArc_euk_tags_ordered_no_tax.txt", h = T, row.names=1)

taxapooler_in_euk_PA_no99 <- taxapooler_in_euk_PA[rowSums(taxapooler_in_euk_PA)>99,]

require(vegan)

taxapooler_in_euk_PA_no99_pa <- decostand(taxapooler_in_euk_PA_no99, method = "pa")

#singletons are excluded

#dist_CORE_bac_jaccard <- vegdist(t(shared_OTU_bac_input), method="jaccard")

#dist_CORE_bac_jaccard <- as.matrix(dist_CORE_bac_jaccard)

#repeat, but remove singletons and <99 reads first

#create distance matrix

shared_OTU_euk_no99_input <- read.table("./euk/shared_OTU_euk_no99_input.txt", h = T, row.names=1)

dist_CORE_no_99_euk_jaccard <- vegdist(t(shared_OTU_euk_no99_input), method="jaccard")

dist_CORE_no_99_euk_jaccard <- as.matrix(dist_CORE_no_99_euk_jaccard)

###Piers script for NMDS and ANOSIM

NMDS_input_euk <- read.table("./euk/NMDS_euk_input2.txt", header=TRUE, row.names=1) #input table with environments in order, removed samples that were replicates

NMDS_input_euk <- t(NMDS_input_euk)

Euk_Groupings <- c(

rep("DSAGG", 8),

rep("HGC", 2),

"Ice",

rep("IceB", 8),

rep("IceBr", 2),

"IceNew",

rep("IceS", 7),

rep("MPAGG", 3),

rep("MPW", 3),

rep("SED", 3),

rep("SW", 7))

Euk_Colours <- c(

rep("olivedrab", 8),

rep("tan1", 2),

"snow",

rep("cadetblue", 8),

rep("aquamarine", 2),

"cornflowerblue",

rep("paleturquoise", 7),

rep("seagreen", 3),

rep("deepskyblue", 3),

rep("burlywood4", 3),

rep("royalblue4", 7))

require(vegan)

#create presence/absence input

NMDS_input_euk_pa <- decostand(NMDS_input_euk, method = "pa")

# Test for different in mean rank between groups...

anosim(NMDS_input_euk, Euk_Groupings, distance = "bray")

anosim(NMDS_input_euk_pa, Euk_Groupings, distance = "jaccard")

#Call:

#anosim(dat = NMDS_input_euk, grouping = Euk_Groupings, distance = "bray")

#Dissimilarity: bray

#ANOSIM statistic R: 0.6334

# Significance: 0.001

#Permutation: free

#Number of permutations: 999

#Call:

#anosim(dat = NMDS_input_euk_pa, grouping = Euk_Groupings, distance = "jaccard")

#Dissimilarity: jaccard

#ANOSIM statistic R: 0.6946

# Significance: 0.001

#Permutation: free

#Number of permutations: 999

# calculate jaccard dissimilarity matrix...

Euk_Dist_jaccard <- vegdist(NMDS_input_euk_pa, distance = "jaccard")

# perform metaMDS routine...

NMDS_euk <- metaMDS(Euk_Dist_jaccard, distance = "jaccard", distmethod= "jaccard") # Run 7 stress 0.1251803

# plot NMDS result...

ordiplot(NMDS_euk, type = "t", display = "sites")

# overlay cluster analysis result (keep the previous plot window open) to

# cross-validate ordination spacing...

ordicluster(NMDS_euk, hclust(Euk_Dist_jaccard, method = "average"), prune = 0, display

= "sites")

# final plot

pdf(

file = "IceArc_euk_NMDS_jaccard.pdf",

paper = "a4",

pointsize = 12,

colormodel = "srgb",

useDingbats = F

)

plot(

NMDS_euk,

type = "n"

)

ordiellipse(

NMDS_euk,

Euk_Groupings,

kind = "se", # standard error

conf = 0.95,

lwd = 2,

draw = "polygon",

col="mistyrose",

border = "mistyrose3"

)

ordispider(

NMDS_euk,

Euk_Groupings,

label = F,

#draw = "lines",

col = "grey"

)

points(

NMDS_euk,

pch = 21,

bg = Euk_Colours,

col = "black",

cex = 1.7

)

dev.off()

# calculate bray curtis dissimilarity matrix...

Euk_Dist_bray <- vegdist(NMDS_input_euk, distance = "bray")

# perform metaMDS routine...

NMDS_euk_bray <- metaMDS(Euk_Dist_bray, distance = "bray", distmethod= "bray") # Run 12 stress 0.1428343

# plot NMDS result...

ordiplot(NMDS_euk_bray, type = "t", display = "sites")

# overlay cluster analysis result (keep the previous plot window open) to

# cross-validate ordination spacing...

ordicluster(NMDS_euk_bray, hclust(Euk_Dist_bray, method = "average"), prune = 0, display

= "sites")

# final plot

pdf(

file = "IceArc_euk_NMDS_bray.pdf",

paper = "a4",

pointsize = 12,

colormodel = "srgb",

useDingbats = F

)

plot(

NMDS_euk_bray,

type = "n"

)

ordiellipse(

NMDS_euk_bray,

Euk_Groupings,

kind = "se", # standard error

conf = 0.95,

lwd = 2,

draw = "polygon",

col="mistyrose",

border = "mistyrose3"

)

ordispider(

NMDS_euk_bray,

Euk_Groupings,

label = F,

#draw = "lines",

col = "grey"

)

points(

NMDS_euk_bray,

pch = 21,

bg = Euk_Colours,

col = "black",

cex = 1.7

)

dev.off()

#check for correlation of both distance measures

Mantel_test_Euk_bray_jaccard=mantel(Euk_Dist_jaccard,Euk_Dist_bray, method="spearman", permutations=1000) # first try with fewer permutations, to see how long it takes

#Call:

#mantel(xdis = Euk_Dist_jaccard, ydis = Euk_Dist_bray, method = "spearman", permutations = 1000)

#Mantel statistic r: 0.8463

# Significance: 0.000999

#Upper quantiles of permutations (null model):

# 90% 95% 97.5% 99%

#0.0996 0.1300 0.1643 0.1995

#Permutation: free

#Number of permutations: 1000

plot(Euk_Dist_jaccard,Euk_Dist_bray)

scatter.smooth(Euk_Dist_jaccard,Euk_Dist_bray,pch=16)

#You can also add a linear model line after you have plotted:

L_class3=lm(Euk_Dist_jaccard ~ Euk_Dist_bray)

summary(L_class3)

#Call:

#lm(formula = Euk_Dist_jaccard ~ Euk_Dist_bray)

#Residuals:

# Min 1Q Median 3Q Max

#-0.224149 -0.040405 -0.002717 0.039743 0.231821

#Coefficients:

# Estimate Std. Error t value Pr(>|t|)

#(Intercept) 0.26995 0.01082 24.95 <2e-16 ***

#Euk_Dist_bray 0.62845 0.01246 50.44 <2e-16 ***

#---

#Signif. codes: 0 '***' 0.001 '**' 0.01 '*' 0.05 '.' 0.1 ' ' 1

#Residual standard error: 0.06348 on 988 degrees of freedom

#Multiple R-squared: 0.7203, Adjusted R-squared: 0.72

#F-statistic: 2544 on 1 and 988 DF, p-value: < 2.2e-16

##test correlation of Bacteria vs Eukaryotes data

Mantel and Procrustes tests in R

#What you need is your two OTU tables (same set of samples) in the same order. The relevant functions are available in the vegan library. For the Mantel test you need to produce dissimilarity matrices, i.e. bray-curtis/jaccard. Those can then be tested as:

Mantel_input_bac <- read.table("./bac/Mantel_bac_input2.txt", header=TRUE, row.names=1) #input table with environments in order, removed samples that were replicates

Mantel_input_bac <- t(Mantel_input_bac)

Mantel_input_euk <- read.table("./euk/Mantel_euk_input2.txt", header=TRUE, row.names=1) #input table with environments in order, removed samples that were replicates

Mantel_input_euk <- t(Mantel_input_euk)

Mantel_input_euk_pa <- decostand(Mantel_input_euk, method ="pa")

Bac_Dist_bray_mantel <- vegdist(Mantel_input_bac, distance = "bray")

Euk_Dist_jaccard_mantel <- vegdist(Mantel_input_euk_pa, distance = "jaccard")

Euk_Dist_bray_mantel <- vegdist(Mantel_input_euk, distance = "bray")

Mantel_test=mantel(Euk_Dist_jaccard_mantel,Bac_Dist_bray_mantel, method="spearman", permutations=1000)

Mantel_test2=mantel(Euk_Dist_bray_mantel,Bac_Dist_bray_mantel, method="spearman", permutations=1000) # first try with fewer permutations, to see how long it takes

#Mantel statistic based on Spearman's rank correlation rho

#Call:

#mantel(xdis = Euk_Dist_jaccard_mantel, ydis = Bac_Dist_bray_mantel, method = "spearman", permutations = 1000)

#Mantel statistic r: 0.8475

# Significance: 0.000999

#Upper quantiles of permutations (null model):

# 90% 95% 97.5% 99%

#0.112 0.144 0.164 0.212

#Permutation: free

#Number of permutations: 1000

#Mantel statistic based on Spearman's rank correlation rho

#Call:

#mantel(xdis = Euk_Dist_bray_mantel, ydis = Bac_Dist_bray_mantel, method = "spearman", permutations = 1000)

#Mantel statistic r: 0.7557

# Significance: 0.000999

#Upper quantiles of permutations (null model):

# 90% 95% 97.5% 99%

#0.106 0.140 0.167 0.208

#Permutation: free

#Number of permutations: 1000

#You can plot it and add a scatter smooth line as follows (probably not needed for the Ms; just the Mantel result will do):

plot(Bac_Dist_bray_mantel,Euk_Dist_jaccard_mantel)

scatter.smooth(Bac_Dist_bray_mantel,Euk_Dist_jaccard_mantel,pch=16)

#You can also add a linear model line after you have plotted:

L_class=lm(Bac_Dist_bray_mantel ~ Euk_Dist_jaccard_mantel)

summary(L_class)

abline(L_class)

#Call:

#lm(formula = Bac_Dist_bray_mantel ~ Euk_Dist_jaccard_mantel)

#Residuals:

# Min 1Q Median 3Q Max

#-0.42633 -0.03293 0.01041 0.04720 0.33628

#Coefficients:

# Estimate Std. Error t value Pr(>|t|)

#(Intercept) 0.19675 0.01832 10.74 <2e-16 ***

#Euk_Dist_jaccard_mantel 0.86009 0.02254 38.16 <2e-16 ***

#---

#Signif. codes: 0 '***' 0.001 '**' 0.01 '*' 0.05 '.' 0.1 ' ' 1

#Residual standard error: 0.08245 on 901 degrees of freedom

#Multiple R-squared: 0.6178, Adjusted R-squared: 0.6174

#F-statistic: 1456 on 1 and 901 DF, p-value: < 2.2e-16

plot(Bac_Dist_bray_mantel,Euk_Dist_bray_mantel)

scatter.smooth(Bac_Dist_bray_mantel,Euk_Dist_bray_mantel,pch=16)

#You can also add a linear model line after you have plotted:

L_class2=lm(Bac_Dist_bray_mantel ~ Euk_Dist_bray_mantel)

summary(L_class2)

abline(L_class2)

#Call:

#lm(formula = Bac_Dist_bray_mantel ~ Euk_Dist_bray_mantel)

#Residuals:

# Min 1Q Median 3Q Max

#-0.54533 -0.02797 0.01738 0.03838 0.33101

#Coefficients:

Estimate Std. Error t value Pr(>|t|)

#(Intercept) 0.40080 0.01607 24.94 <2e-16 ***

#Euk_Dist_bray_mantel 0.57227 0.01853 30.88 <2e-16 ***

#---

#Signif. codes: 0 '***' 0.001 '**' 0.01 '*' 0.05 '.' 0.1 ' ' 1

#Residual standard error: 0.09295 on 901 degrees of freedom

#Multiple R-squared: 0.5142, Adjusted R-squared: 0.5137

#F-statistic: 953.8 on 1 and 901 DF, p-value: < 2.2e-16

##### create bar plot on different taxonomic resolution

require(vegan)

IceArc_tags_euk_in <- read.table("X_IceArc_euk_tags_ordered_taxpoolerIn.txt", head=T, row.names=1)

source("taxa.pooler.1.4.r")

taxapooler_IceArc_euk <-taxa.pooler(IceArc_tags_euk_in) #45 samples in order, 8 tax levels

#manually calculated relative abundance of taxa per taxonomic level

##Plot Bar chart most abundant

#on genus level

relData_euk_genus <- read.table("./euk/genus.matrix_rel.txt", header=TRUE, row.names=1)

relData_euk_genus = t(relData_euk_genus)

source("PlotAbund_test2.R")

IceArc_euk_genus_top5 <- PlotAbund(relData_euk_genus, 5) #plot only top 5 most abundant taxa

#on class level

relData_class <- read.table("TAX.class.matrix_2_rel.txt", header=TRUE, row.names=1)

relData_class = t(relData_class)

source("PlotAbund_test2.R")

IceArc_class_top5 <- PlotAbund(relData_class, 5)

#generate species accumulation curves

require(vegan)

#e.g. for sea ice surface communities:

###IceS

phylum_euk_IceS <- read.table("./euk/spec_accu_phylum_IceS.txt", h=TRUE, row.names=1)

spec_phylum_euk_IceS=specaccum(phylum_euk_IceS, method="random")

boxplot(spec_phylum_euk_IceS)

summary(spec_phylum_euk_IceS)

1 sites 2 sites 3 sites 4 sites 5 sites 6 sites 7 sites

Min. :13.00 Min. :17.00 Min. :20.00 Min. :22.00 Min. :23.0 Min. :25.00 Min. :27

1st Qu.:15.00 1st Qu.:19.00 1st Qu.:21.00 1st Qu.:23.00 1st Qu.:25.0 1st Qu.:26.00 1st Qu.:27

Median :16.00 Median :20.00 Median :23.00 Median :25.00 Median :26.0 Median :27.00 Median :27

Mean :16.47 Mean :20.60 Mean :22.68 Mean :24.41 Mean :25.5 Mean :26.34 Mean :27

3rd Qu.:17.00 3rd Qu.:22.25 3rd Qu.:24.00 3rd Qu.:25.00 3rd Qu.:27.0 3rd Qu.:27.00 3rd Qu.:27

Max. :21.00 Max. :25.00 Max. :27.00 Max. :27.00 Max. :27.0 Max. :27.00 Max. :27

#generate output for all habitats on the desired taxonomic level

#generate merged plot, displaying all curves together

pdf(

file = "Spec_accum_euk_IceS.pdf",

paper = "a4",

pointsize = 12,

colormodel = "srgb",

useDingbats = F

)

boxplot(spec_genus_euk_IceS)#, add=TRUE, border="red")

boxplot(spec_family_euk_IceS, add=TRUE, border="cornflowerblue")

boxplot(spec_order_euk_IceS, add=TRUE, border="olivedrab")

boxplot(spec_class_euk_IceS, add=TRUE, border="orange")

boxplot(spec_phylum_euk_IceS, add=T, border="darkred")

#legend(10,370, c("Phylum", "Class", "Order", "Family", "Genus", "Species"),fill = c("red", "blue","grey","orange","black", "green" ),cex=1.2)

legend(6,170, c("Genus", "Family", "Order", "Class", "Phylum"),fill = c("black", "cornflowerblue","olivedrab","orange","darkred"),cex=1.2)

dev.off()
