# Supplementary material for: Effects of Ice-Algal Aggregate Export on the Connectivity of Bacterial Communities in the Central Arctic Ocean
Source: Front Microbiol. 2018 May 18;9:1035. doi: 10.3389/fmicb.2018.01035 (PMC5974969; doi:10.3389/fmicb.2018.01035)
Supplement: Supplementary file 4 [file Data_Sheet_3.pdf]

# *Supplementary Material*

# Effects of Ice-Algal Aggregate Export on the Connectivity of Bacterial Communities in the Central Arctic Ocean

**Josephine Z. Rapp[1, 2*], Mar Fernández-Méndez[3], Christina Bienhold[1, 2] & Antje Boetius[1, 2, 4]**

*** Correspondence:**
Josephine Z. Rapp
josephine.rapp@awi.de

## 1 Supplementary Information

## 1.1 Material and Methods

### 1.1.1 Automated Ribosomal Intergenic Spacer Analysis (ARISA)

To corroborate the observed bacterial community patterns, we used Automated Ribosomal Intergenic Spacer Analysis (Fisher and Triplett, 1999) in parallel to the tag sequencing approach. Following a similar approach, we covered all nine stations, with one sample from sea-ice surface and bottom, water column and pooled sediment samples. A larger number of samples from individual melt ponds, sea-ice algal aggregates and holothurian digestive tract content was available for ARISA, leaving us with a total number of 68 different samples.

The PCR mix for the ITS region contained 2.5 µl of 10x reaction buffer S (PEQLAB Biotechnologie GmbH, Erlangen, Germany), 1 µl of 25 mM MgCl2 (PEQLAB Biotechnologie GmbH, Erlangen, Germany), 0.625 µl of a 10 mM dNTP mix (PEQLAB Biotechnologie GmbH, Erlangen, Germany), 0.75 µl of 3 mg ml-1 bovine serum albumin (Sigma-Aldrich Chemie Gmbh; Munich, Germany), 0.25 µl of 40 µM universal forward primer ITSF (5'- GTCGTAACAAGGTAGCCGTA-3') (Biomers.net, Ulm, Germany), labeled with 6- carboxyfluorescein (FAM), 0.25 µl of 40 µM ITSReub reverse primer (5'- GCCAAGGCATCCACC-3') (Biomers.net, Ulm, Germany), 0.25 µl of 5 units µl-1 Taq polymerase (PEQLAB Biotechnologie GmbH, Erlangen, Germany) and approximately 10 ng environmental DNA. PCR water was added to each reaction mix to a final volume of 25 µl. Additionally, control reactions with either no template DNA (negative), or with DNA from sediment sampled on Sylt, Germany, (positive) were performed. All reactions were conducted in triplicates. The PCR was carried out in an Eppendorf MasterCycler (Eppendorf AG, Hamburg, Germany). After an initial denaturation for 3 min at 94°C, 30 cycles of first 94°C for 45 sec, 55°C for 45 sec and 72°C for 90 sec followed. Final extension time was 5 min at 72°C. PCR products were checked on a 1.5% agarose gel (3 g of LE agarose (Biozym Scientific GmbH, Hessisch Oldendorf, Germany) in 200 ml of 1x TAE (Tris-acetate-EDTA)-buffer), and PCR products were purified using SephadexTM G-50

Superfine (GE Healthcare, Bio-Sciences AB, Uppsala, Sweden) to remove low molecular substances and other contaminants.

Fragment analysis of the PCR products was done via capillary electrophoresis. A standardized amount of 100 ng amplified DNA was mixed with 0.5 µl of the internal size standard MapMarker® 1000, labeled with ROX (BioVentures, Inc. Murfreesboro, TN, USA), and 14 µl of deionized HiDiTM formamide (Applied Biosystems, California, USA). Samples were denatured at 95°C for 3 min (Eppendorf Mastercycler gradient, Eppendorf, Hamburg, Germany) and immediately put on ice for 5 min. Capillary electrophoresis was carried out on an ABI Prism 3130 XL - Genetic Analyzer (Applied Biosystems, California, USA).

#### 1.1.1.1 ARISA Statistical analysis

Electropherograms were analyzed using the GeneMapper Software v3.7 (Applied Biosystems, California, USA). Subsequent binning of peaks was performed in R (R-Project; R Foundation for Statistical Computing Version 2.14.0; R Development Core Team, 2011, http://www.R-project.org), using a custom R script (Interactive Binner function, www.mpi-bremen.de/en/Software-4.html#section1549) (Ramette 2009) and a window size of 2 base pairs (bp) to compensate for slight technical shifts between profiles and fragment size calling imprecision. Only fragments with a length between 100 bp and 1000 bp and peaks with ≥50 relative fluorescence units and ≥0.09% relative fluorescence intensity (individual peak areas divided by the total peak area of the respective sample) were considered. Replicate PCR profiles were merged, using a custom R script (https://www.mpi-bremen.de/Binaries/Binary1658/replicate-merger-ALk-consensus-RFI-1.2.r) and $OTU_{ARISA}$ were considered present if appearing in at least two of the three PCR replicates.

## 1.2 Results

### 1.2.1 ARISA Results

Analysis of the bacterial community structure revealed differences between the investigated environments. NMDS showed an apparent clustering of samples from the sea-ice environment (including all ice and melt pond samples), the water column (including all surface seawater and water under the ice samples) and the deep sea (including all samples from deep-sea sediments, holothurian digestive tract content and deposited aggregates), when grouping the data set a posteriori according to the different environments (Figure S8). ANOSIM confirmed significant differences of bacterial community structure between the sea-ice and the deep-sea environments (R=0.81; Bonferroni-corrected p=0.003), and even more pronounced structural differences between the water column and the deep-sea environment (R=0.99; Bonferroni-corrected p=0.003). Sea-ice bacterial community structure was highly dissimilar from any of the other investigated environments, but appeared host communities partially overlapping with those found in the water column (R=0.63; Bonferroni-corrected p=0.003) and in melt ponds (R= 0.41; Bonferroni-corrected p=0.003).

All, sea ice, melt ponds and surface seawater, showed strongest structural differences to the deep-sea environments. The overall observed bacterial community pattern using NMDS on the ARISA results resembled community patterns observed with Illumina tag sequencing (Figure 4 & Figure S8), and dissimilarity matrices from both methods were significantly correlated (Figure S9).

## 1.3 Discussion

The observed OTU richness in ice-associated environments and surface seawater, both for bacteria and eukaryotes (Figure 2), was in the range of results reported from previous Arctic molecular surveys based on next- generation sequencing of the SSU rRNA genes (Bowman et al., 2012; Kilias et al., 2014a, 2014b; Meshram et al., 2017; Stecher et al., 2016; Thaler and Lovejoy, 2015), but lower than numbers reported from Arctic coastal regions and marginal seas (Comeau et al., 2013; Ghiglione et al., 2012; Hatam et al., 2014, 2016). OTU richness in sediments was also comparable to, but at the lower end of OTU numbers reported for sediments from the Siberian continental margin (Bienhold et al., 2012) and the Fram Strait (Jacob et al., 2013). The wide Arctic shelves have a higher nutrient availability (Le Fouest et al., 2013; Garneau et al., 2006) and sustain higher productivity and microbial standing stocks (Tremblay et al., 2011), and it is therefore likely that they allow for higher diversity than the oligotrophic central basins (Horner-Devine et al., 2003).

### 1.3.1 Connectivity of microbial communities in the central Arctic Ocean and potential effects of climate change

Bacterial generalists, represented by members of the genera *Colwellia*, *Oleispira* and *Lentimonas*, showed very high sequence numbers in ice-associated environments, water column and ice-algae deposits, and low proportions in sediment. This may indicate that they also originate from surface environments, and are not indigenous benthic bacteria. The genus *Colwellia* exhibits distinct

adaptations to life at low temperatures (Huston et al., 2004) and has been found in a range of environments, including sea ice (Bowman et al., 1997), deep-sea sediment (Deming et al., 1988) and associated to particles or algae in seawater (Bowman et al., 1998). Its capability to degrade high-molecular-weight organic compounds suggests an important role of this genus in carbon and nutrient cycling in cold environments (Methe et al., 2005). Similarly, *Oleispira* contains several cold-adaptations in its genome (Kube et al., 2013) and its presence was reported from polar seawater and the deep sea (Li et al., 2015; Yakimov et al., 2003). Interestingly, this genus is part of an ecophysiologically unusual group of bacteria whose metabolism is restricted to the degradation of hydrocarbons, and its presence has so far always been attributed to environments contaminated with crude oil (Yakimov et al., 2003); their role in the Arctic environments sampled here thus remains to be determined. Very little is known about the genus *Lentimonas*. Its presence has been reported for seawater and sediment (Freitas et al., 2012), and its close relatives from the *Verrucomicrobia* phylum are known for their capacity to degrade highly complex polysaccharide substrates (Cardman et al., 2014; Martinez-Garcia et al., 2012), therefore suggesting a potential role of *Lentimonas* in organic matter cycling. Bacterial groups transported with the algal aggregates may thus locally influence carbon turnover of the aggregates at the seafloor, yet their persistence and contribution to the degradation and recycling of organic material remains unclear until temporal observations and measures of activity become available. The representation of surface-derived cells in the sediment may potentially increase after repeated deposition events or longer exposure of the aggregates at the seafloor (Kellogg and Deming, 2009).

## References

Bienhold, C., Boetius, A., and Ramette, A. (2012). The energy-diversity relationship of complex bacterial communities in Arctic deep-sea sediments. *ISME J.* 6, 724–32. doi:10.1038/ismej.2011.140.

Bowman, J. P., Brown, M. V., and Nichols, D. S. (1997). Biodiversity and ecophysiology of bacteria associated with Antarctic sea ice. *Antarct. Sci.* 9, 134–142. doi:10.1017/S0954102097000175.

Bowman, J. P., Gosink, J. J., McCammon, S. A., Lewis, T. E., Nichols, D. S., Nichols, P. D., et al. (1998). Colwellia demingiae sp. nov., Colwellia hornerae sp. nov., Colwellia rossensis sp. nov. and Colwellia psychrotropica sp. nov.: psychrophilic Antarctic species with the ability to synthesize docosahexaenoic acid (22: 63). *Int. J. Syst. Bacteriol.* 48, 1171–1180. doi:10.1099/00207713-48-4-1171.

Bowman, J. S., Rasmussen, S., Blom, N., Deming, J. W., Rysgaard, S., and Sicheritz-Ponten, T. (2012). Microbial community structure of Arctic multiyear sea ice and surface seawater by 454 sequencing of the 16S RNA gene. *ISME J.* 6, 11–20. doi:10.1038/ismej.2011.76.

Cardman, Z., Arnosti, C., Durbin, A., Ziervogel, K., Cox, C., Steen, A. D., et al. (2014). Verrucomicrobia are candidates for polysaccharide-degrading bacterioplankton in an Arctic fjord of Svalbard. *Appl. Environ. Microbiol.* 80, 3749–3756. doi:10.1128/AEM.00899-14.

Comeau, A. M., Philippe, B., Thaler, M., Gosselin, M., Poulin, M., and Lovejoy, C. (2013). Protists in Arctic drift and land-fast sea ice. *J. Phycol.* 49, 229–240. doi:10.1111/jpy.12026.

Deming, J. W., Somers, L. K., Straube, W. L., Swartz, D. G., and Macdonell, M. T. (1988). Isolation of an Obligately Barophilic Bacterium and Description of a New Genus, Colwellia gen. nov. *Syst. Appl. Microbiol.* 10, 152–160. doi:10.1016/S0723-2020(88)80030-4.

Fisher, M. M., and Triplett, E. W. (1999). Automated approach for ribosomal intergenic spacer analysis of microbial diversity and its application to freshwater bacterial communities. *Appl. Environ. Microbiol.* 65, 4630–4636. Available at: http://www.ncbi.nlm.nih.gov/pubmed/10508099 [Accessed November 5, 2017].

Le Fouest, V., Babin, M., and Tremblay, J. E. (2013). The fate of riverine nutrients on Arctic shelves. *Biogeosciences* 10, 3661–3677. doi:10.5194/bg-10-3661-2013.

Freitas, S., Hatosy, S., Fuhrman, J. A., Huse, S. M., Welch, D. B. M., Sogin, M. L., et al. (2012). Global distribution and diversity of marine Verrucomicrobia. *ISME J.* 6, 1499–1505. doi:10.1038/ismej.2012.3.

Garneau, M.-ève, Vincent, W. F., Alonso-sáez, L., Gratton, Y., and Lovejoy, C. (2006). Prokaryotic community structure and heterotrophic production in a river-influenced coastal arctic ecosystem. 42, 27–40.

Ghiglione, J.-F., Galand, P. E., Pommier, T., Pedrós-Alió, C., Maas, E. W., Bakker, K., et al. (2012). Pole-to-pole biogeography of surface and deep marine bacterial communities. *Proc. Natl. Acad. Sci. U. S. A.* 109, 17633–8. doi:10.1073/pnas.1208160109.

Hatam, I., Charchuk, R., Lange, B., Beckers, J., Haas, C., and Lanoil, B. (2014). Distinct bacterial assemblages reside at different depths in Arctic multiyear sea ice. *FEMS Microbiol. Ecol.* 90, 115–125. doi:10.1111/1574-6941.12377.

Hatam, I., Lange, B., Beckers, J., Haas, C., and Lanoil, B. (2016). Bacterial communities from Arctic seasonal sea ice are more compositionally variable than those from multi-year sea ice. *ISME J.* 10, 2543–2552. doi:10.1038/ismej.2016.4.

Horner-Devine, M. C., Leibold, M. A., Smith, V. H., and Bohannan, B. J. M. (2003). Bacterial diversity patterns along a gradient of primary productivity. *Ecol. Lett.* 6, 613–622. doi:10.1046/j.1461-0248.2003.00472.x.

Huston, A. L., Methe, B., and Deming, J. W. (2004). Purification, characterization, and sequencing of an extracellular cold-active aminopeptidase produced by marine psychrophile Colwellia psychrerythraea strain 34H. *Appl. Environ. Microbiol.* 70, 3321–3328. doi:10.1128/AEM.70.6.3321-3328.2004.

Jacob, M., Soltwedel, T., Boetius, A., and Ramette, A. (2013). Biogeography of Deep-sea benthic bacteria at regional scale (LTER HAUSGARTEN, Fram Strait, Arctic). *PLoS One* 8, e72779. doi:10.1371/journal.pone.0072779.

Kellogg, C., and Deming, J. (2009). Comparison of free-living, suspended particle, and aggregate-associated Bacterial and Archaeal communities in the Laptev Sea. *Aquat. Microb. Ecol.* 57, 1–

18. doi:10.3354/ame01317.

Kilias, E., Kattner, G., Wolf, C., Frickenhaus, S., and Metfies, K. (2014a). A molecular survey of protist diversity through the central Arctic Ocean. *Polar Biol.* 37, 1271–1287. doi:10.1007/s00300-014-1519-5.

Kilias, E. S., Peeken, I., and Metfies, K. (2014b). Insight into protist diversity in Arctic sea ice and melt-pond aggregate obtained by pyrosequencing. *Polar Res.* 33, 23466. doi:10.3402/polar.v33.23466.

Kube, M., Chernikova, T. N., Al-Ramahi, Y., Beloqui, A., Lopez-Cortez, N., Guazzaroni, M.-E., et al. (2013). Genome sequence and functional genomic analysis of the oil-degrading bacterium Oleispira antarctica. *Nat. Commun.* 4, 2156. doi:10.1038/ncomms3156.

Li, Y., Liu, Q., Li, C., Dong, Y., Zhang, W., Zhang, W., et al. (2015). Bacterial and archaeal community structures in the Arctic deep-sea sediment. *Acta Oceanol. Sin.* 34, 93–113. doi:10.1007/s13131-015-0624-9.

Martinez-Garcia, M., Brazel, D. M., Swan, B. K., Arnosti, C., Chain, P. S. G., Reitenga, K. G., et al. (2012). Capturing single cell genomes of active polysaccharide degraders: An unexpected contribution of verrucomicrobia. *PLoS One* 7, e35314. doi:10.1371/journal.pone.0035314.

Meshram, A. R., Vader, A., Kristiansen, S., and Gabrielsen, T. M. (2017). Microbial Eukaryotes in an Arctic Under-Ice Spring Bloom North of Svalbard. *Front. Microbiol.* 8, 1099. doi:10.3389/fmicb.2017.01099.

Methe, B. A., Nelson, K. E., Deming, J. W., Momen, B., Melamud, E., Zhang, X., et al. (2005). The psychrophilic lifestyle as revealed by the genome sequence of Colwellia psychrerythraea 34H through genomic and proteomic analyses. *Proc. Natl. Acad. Sci.* 102, 10913–10918. doi:10.1073/pnas.0504766102.

Stecher, A., Neuhaus, S., Lange, B., Frickenhaus, S., Beszteri, B., Kroth, P. G., et al. (2016). rRNA and rDNA based assessment of sea ice protist biodiversity from the central Arctic Ocean. *Eur. J. Phycol.* 51, 31–46. doi:10.1080/09670262.2015.1077395.

Thaler, M., and Lovejoy, C. (2015). Biogeography of heterotrophic flagellate populations indicates the presence of generalist and specialist taxa in the Arctic Ocean. *Appl. Environ. Microbiol.* 81, 2137–2148. doi:10.1128/AEM.02737-14.

Tremblay, J.-É., Bélanger, S., Barber, D. G., Asplin, M., Martin, J., Darnis, G., et al. (2011). Climate forcing multiplies biological productivity in the coastal Arctic Ocean. *Geophys. Res. Lett.* 38, n/a-n/a. doi:10.1029/2011GL048825.

Yakimov, M. M., Giuliano, L., Gentile, G., Crisafi, E., Chernikova, T. N., Abraham, W. R., et al. (2003). Oleispira antarctica gen. nov., sp. nov., a novel hydrocarbonoclastic marine bacterium isolated from Antarctic coastal sea water. *Int. J. Syst. Evol. Microbiol.* 53, 779–785. doi:10.1099/ijs.0.02366-0.
