# Supplementary material for: Effects of Ice-Algal Aggregate Export on the Connectivity of Bacterial Communities in the Central Arctic Ocean
Source: Front Microbiol. 2018 May 18;9:1035. doi: 10.3389/fmicb.2018.01035 (PMC5974969; doi:10.3389/fmicb.2018.01035)
Supplement: Supplementary file 5 [file Image_1.pdf]

Supplementary Material

Effects of Ice-Algal Aggregate Export on the Connectivity of Bacterial Communities in the Central Arctic Ocean

Josephine Z. Rapp[1, 2*], Mar Fernández-Méndez[3], Christina Bienhold[1, 2] & Antje Boetius[1, 2, 4]

*** Correspondence:**
Josephine Z. Rapp
josephine.rapp@awi.de

1 Supplementary Figures

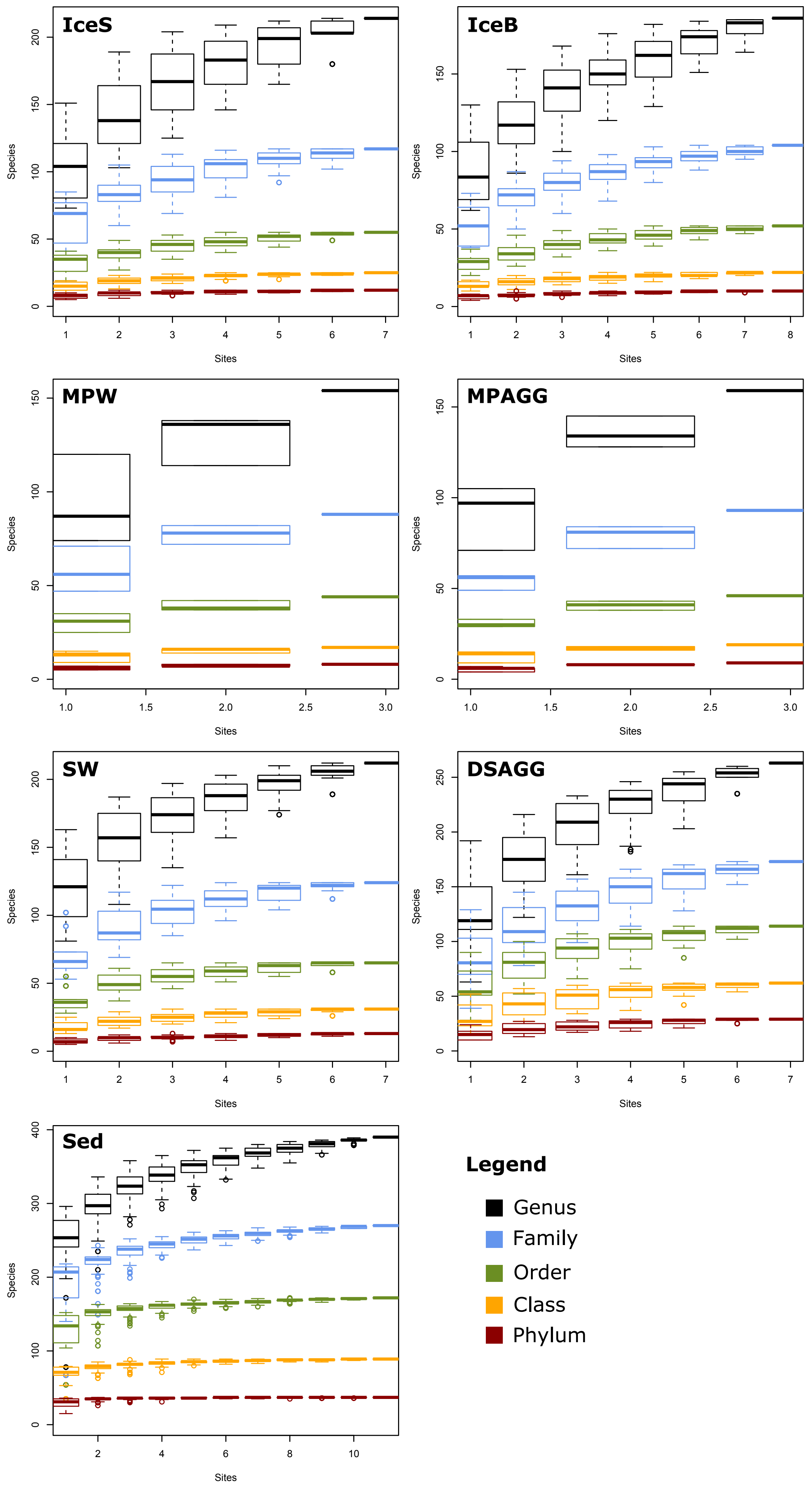

Figure S1 - Accumulation curves per taxonomic level for bacterial communities.

IceS: sea-ice surface; IceB: sea-ice bottom; MPW: melt-pond water; MPAGG: melt-pond aggregate; SW: surface seawater; DSAGG: deep-sea algae deposit; Sed: deep-sea surface sediment.

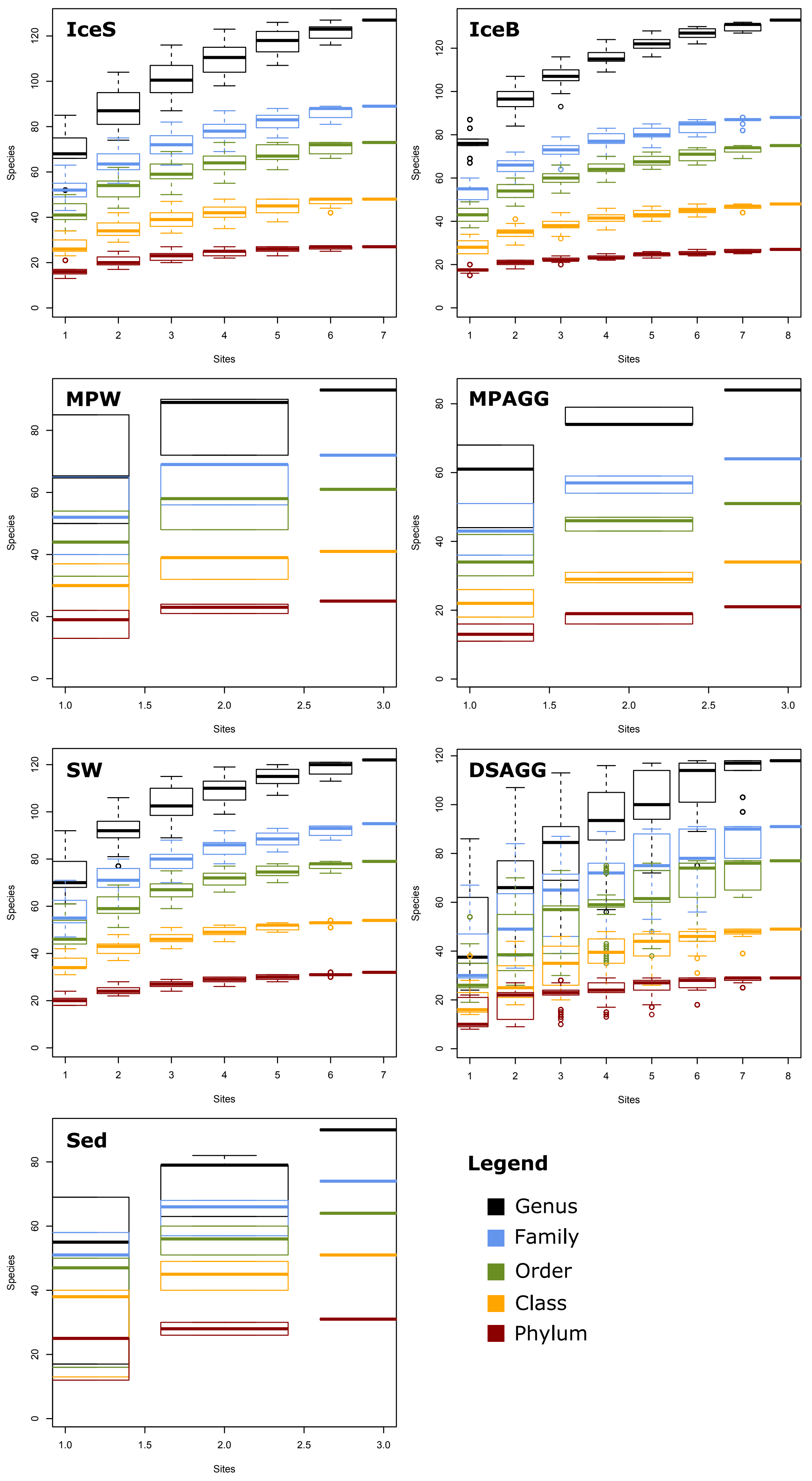

Figure S2 - Accumulation curves per taxonomic level for eukaryotic communities.
IceS: sea-ice surface; IceB: sea-ice bottom; MPW: melt-pond water; MPAGG: melt-pond aggregate; SW: surface seawater; DSAGG: deep-sea algae deposit; Sed: deep-sea surface sediment.

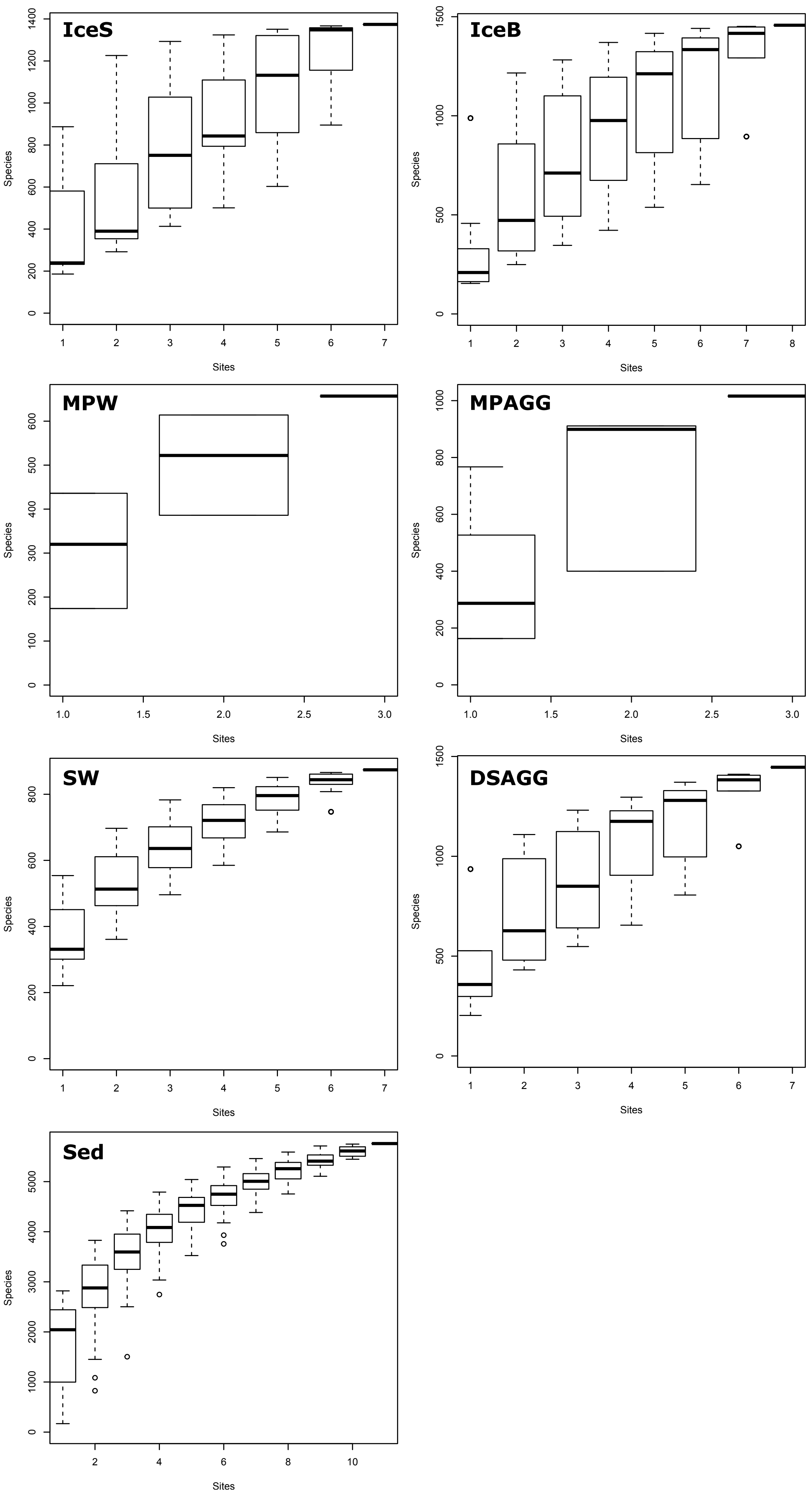

Figure S3 - OTU accumulation curves for bacterial communities.

IceS: sea-ice surface; IceB: sea-ice bottom; MPW: melt-pond water; MPAGG: melt-pond aggregate; SW: surface seawater; DSAGG: deep-sea algae deposit; Sed: deep-sea surface sediment.

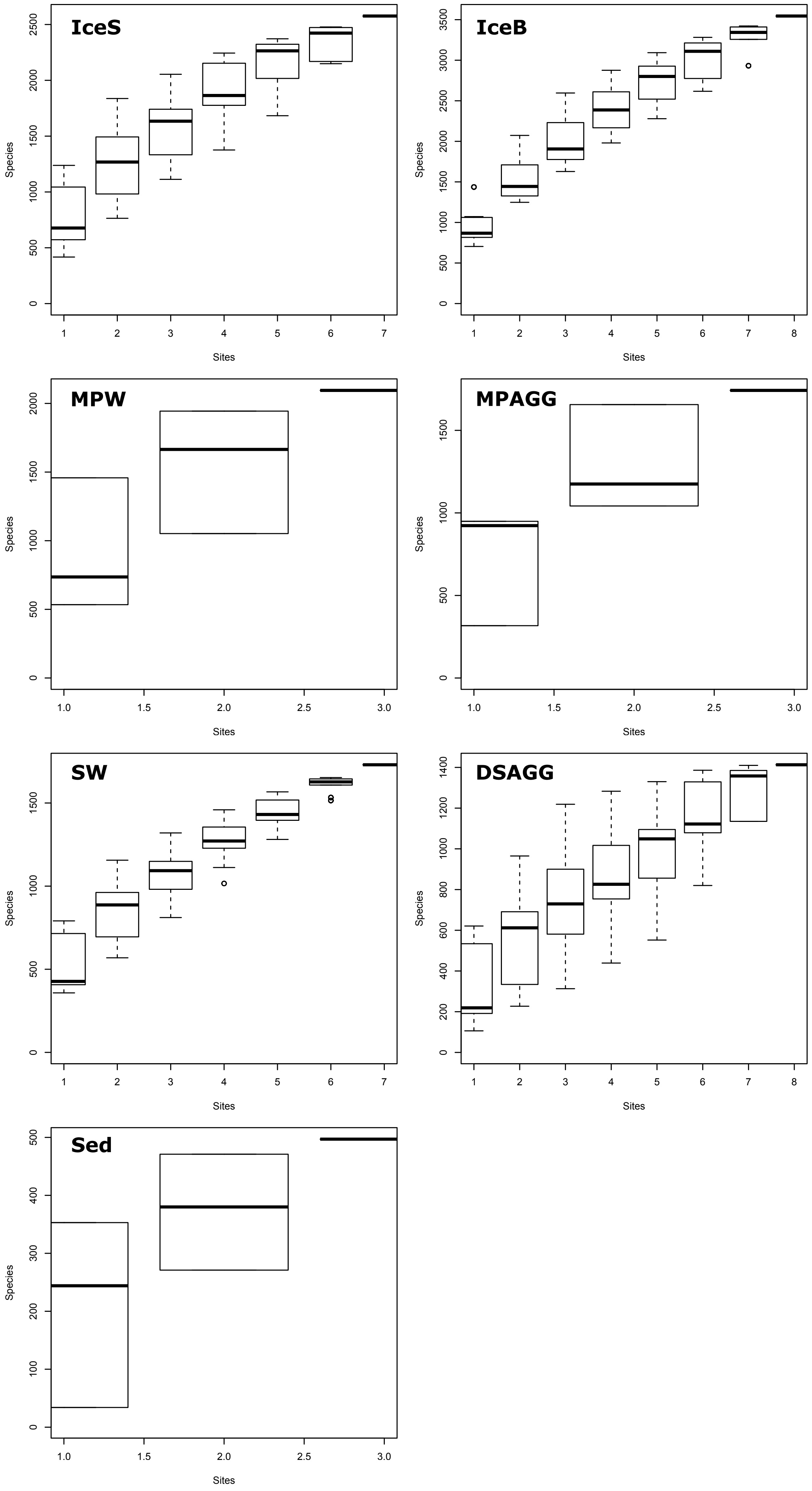

Figure S4 - OTU accumulation curves for eukaryotic communities.

IceS: sea-ice surface; IceB: sea-ice bottom; MPW: melt-pond water; MPAGG: melt-pond aggregate; SW: surface seawater; DSAGG: deep-sea algae deposit; Sed: deep-sea surface sediment.

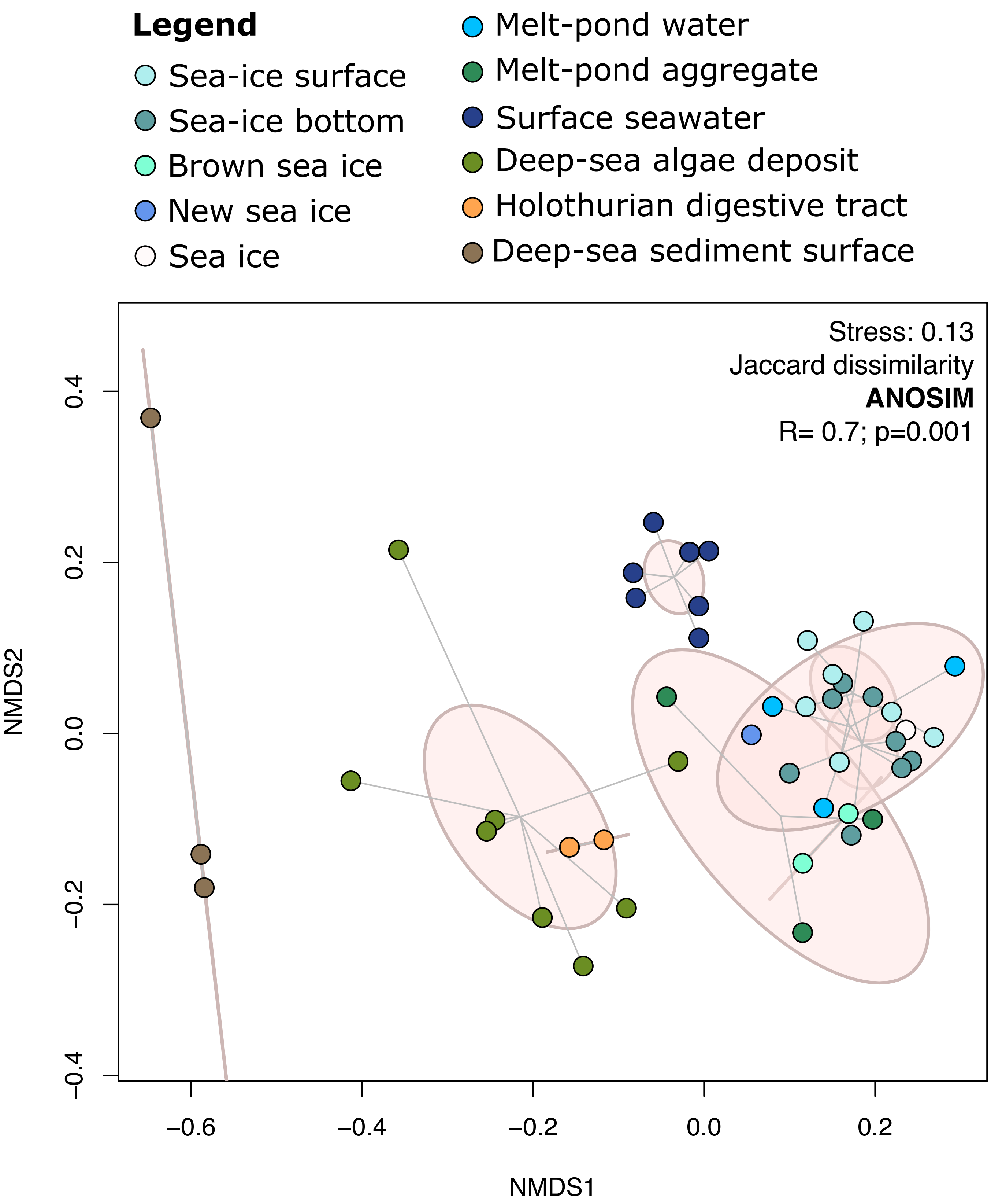

Figure S5 - Two-dimensional NMDS ordination of community dissimilarities for eukaryotes. Dissimilarity matrices and ANOSIM were calculated using the Jaccard dissimilarity measure on the basis of presence/absence of Illumina OTUs. Environments are depicted by color coding and points within each environmental grouping are connected to their group centroid through a spider diagram. Pink ellipses indicate the estimated 95% dispersion limits of each group.

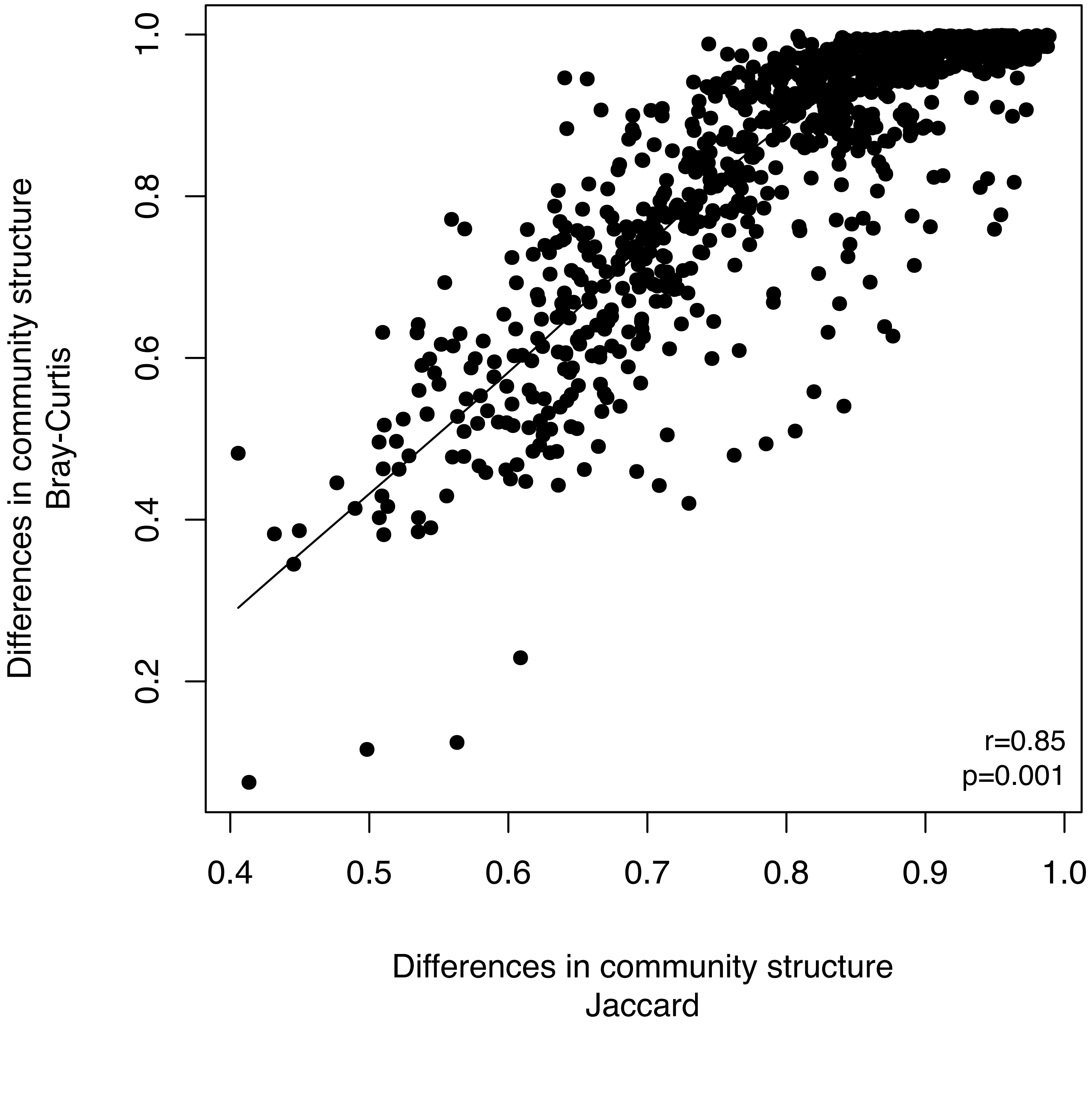

Figure S6 - Correlation of differences in eukaryotic community structure based on the Bray-Curtis and the Jaccard dissimilarity measure for the Illumina OTU dataset. Spearman's correlation as tested by a Mantel test with 999 permutations is indicated in the plot. The black line is a scatter smooth curve computed by LOESS.

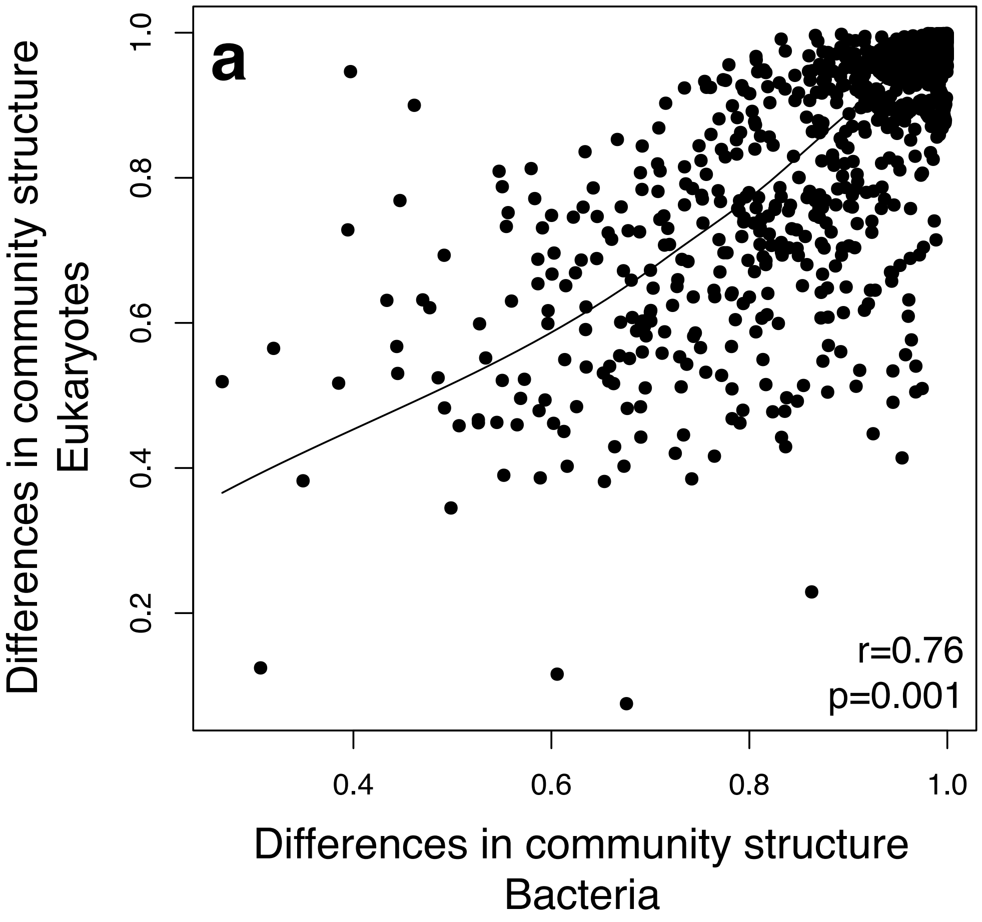

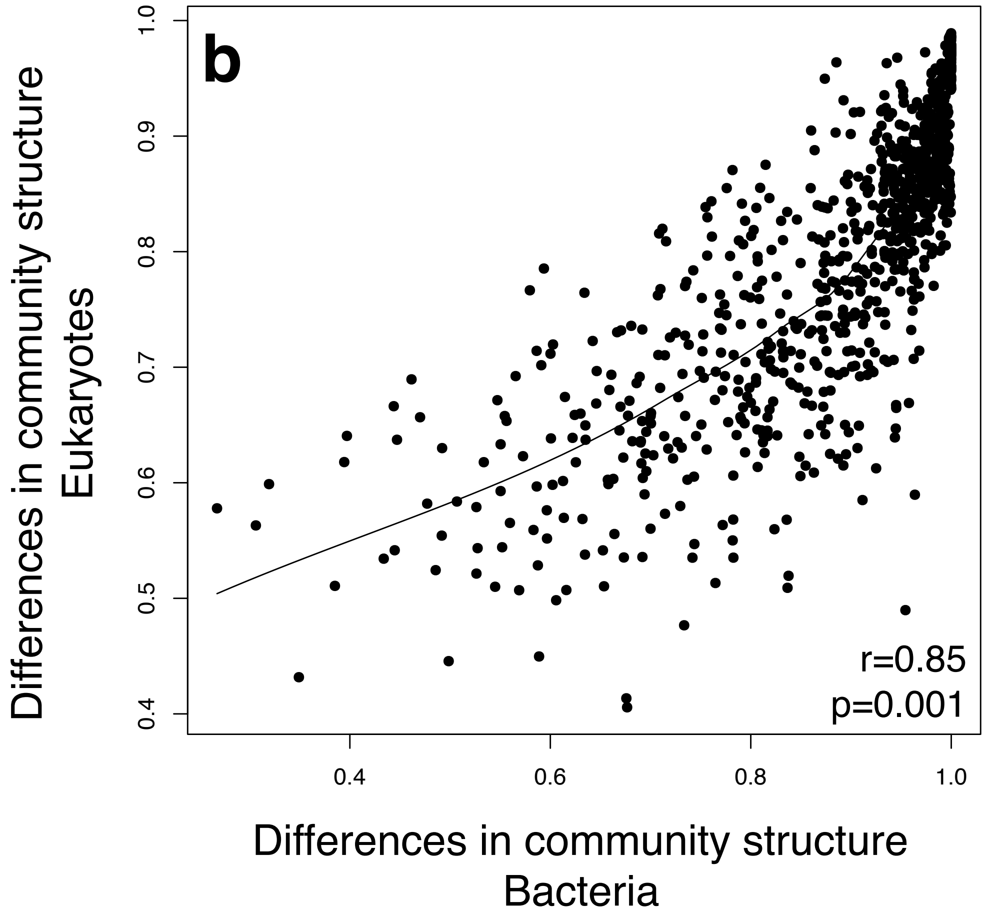

Figure S7 - Correlation of differences in bacterial and eukaryotic community structure based on Illumina OTU datasets. Dissimilarity matrices were calculated using (**a**) the Bray-Curtis dissimilarity measure for both bacteria and eukaryotes and (**b**) the Bray-Curtis dissimilarity measure for bacteria and the Jaccard dissimilarity measure for eukaryotes. Spearman's correlation as tested by a Mantel test with 999 permutations is indicated in the plot. The black line is a scatter smooth curve computed by LOESS.

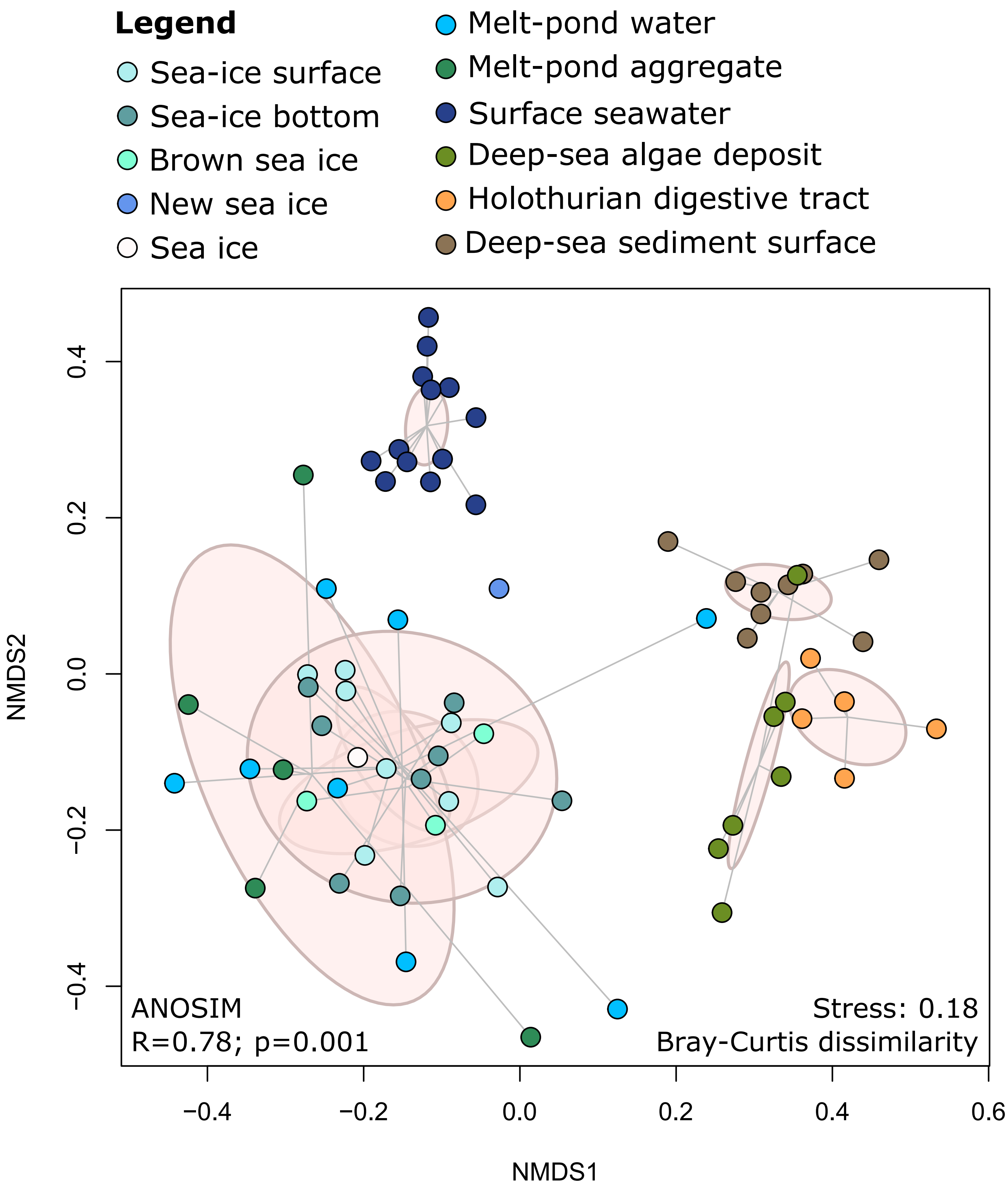

Figure S8 - Two-dimensional NMDS ordination of community dissimilarities for bacteria on the basis of ARISA OTUs. Dissimilarity matrices and ANOSIM were calculated using the Bray-Curtis dissimilarity measure. Environments are depicted by color coding and points within each environmental grouping are connected to their group centroid through a spider diagram. Pink ellipses indicate the estimated 95% dispersion limits of each group.

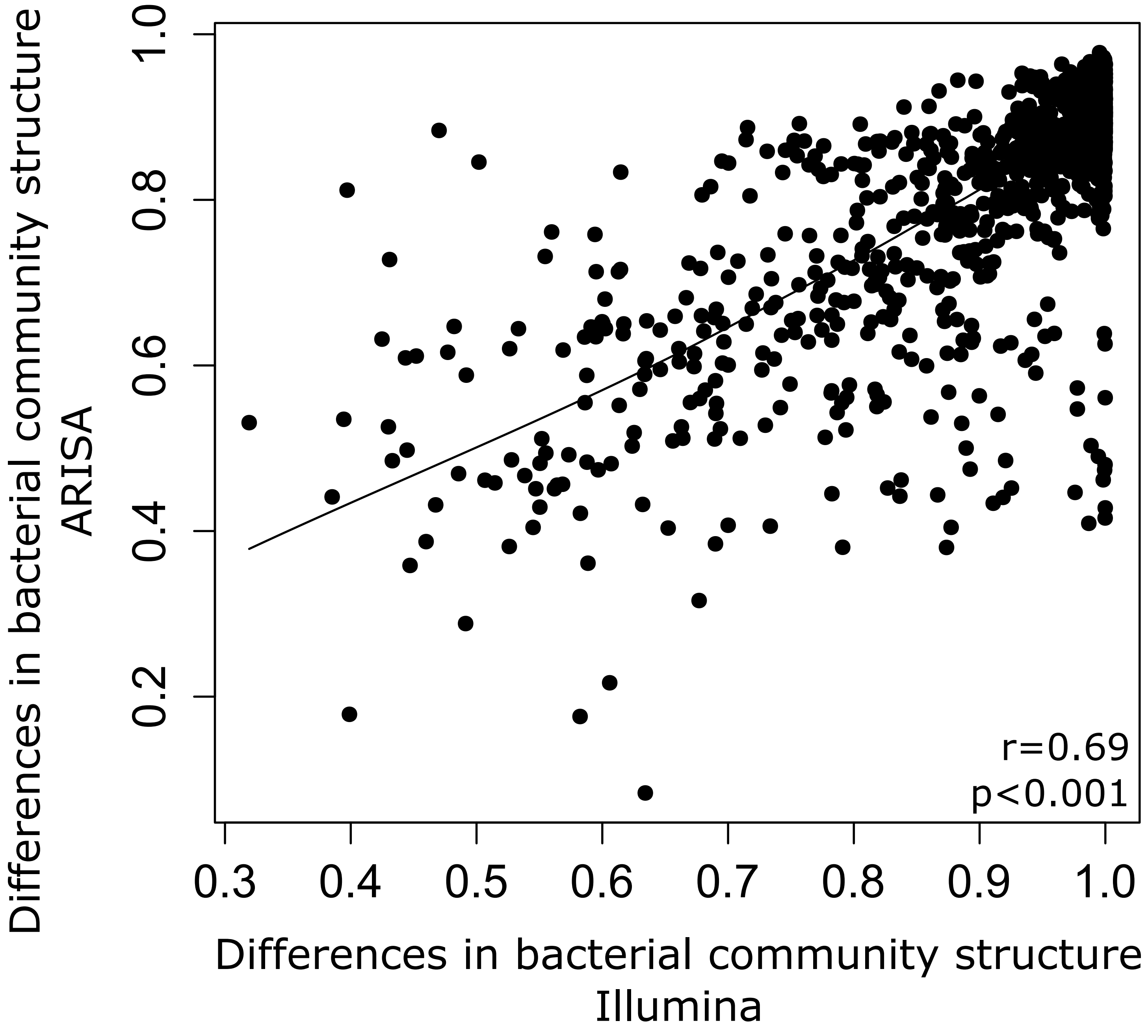

Figure S9 - Correlation of differences in bacterial community structure based on Illumina versus ARISA OTU datasets. Dissimilarity matrices for both datasets were calculated using the Bray-Curtis dissimilarity measure. Spearman's correlation as tested by a Mantel test with 999 permutations is indicated in the plot. The black line is a scatter smooth curve computed by LOESS.
